# Considering Transposable Element Diversification in *De Novo* Annotation Approaches

**DOI:** 10.1371/journal.pone.0016526

**Published:** 2011-01-31

**Authors:** Timothée Flutre, Elodie Duprat, Catherine Feuillet, Hadi Quesneville

**Affiliations:** 1 Unité de Recherche en Génomique-Info, UR 1164, INRA Centre de Versailles-Grignon, Versailles, France; 2 Institut de Minéralogie et de Physique des Milieux Condensés, UMR 7590, CNRS-UPMC-IPGP-Université Paris Diderot, Paris, France; 3 Génétique, Diversité et Ecophysiologie des Céréales, UMR 1095, INRA Domaine du Crouël, Clermont-Ferrand, France; University of Georgia, United States of America

## Abstract

Transposable elements (TEs) are mobile, repetitive DNA sequences that are almost ubiquitous in prokaryotic and eukaryotic genomes. They have a large impact on genome structure, function and evolution. With the recent development of high-throughput sequencing methods, many genome sequences have become available, making possible comparative studies of TE dynamics at an unprecedented scale. Several methods have been proposed for the *de novo* identification of TEs in sequenced genomes. Most begin with the detection of genomic repeats, but the subsequent steps for defining TE families differ. High-quality TE annotations are available for the *Drosophila melanogaster* and *Arabidopsis thaliana* genome sequences, providing a solid basis for the benchmarking of such methods. We compared the performance of specific algorithms for the clustering of interspersed repeats and found that only a particular combination of algorithms detected TE families with good recovery of the reference sequences. We then applied a new procedure for reconciling the different clustering results and classifying TE sequences. The whole approach was implemented in a pipeline using the REPET package. Finally, we show that our combined approach highlights the dynamics of well defined TE families by making it possible to identify structural variations among their copies. This approach makes it possible to annotate TE families and to study their diversification in a single analysis, improving our understanding of TE dynamics at the whole-genome scale and for diverse species.

## Introduction

Transposable elements (TEs) are DNA sequences that can move and duplicate, autonomously or with the assistance of other elements, within genomes. TEs have been described as the “ultimate parasite”, because of their ability to amplify and invade genomes for their own ends [1], and as “selfish DNA sequences” [2]. These invasion events play a particularly important role in eukaryotic genomes, probably because of the smaller population sizes of eukaryotes than of prokaryotes [3].

TEs are generally classified according to their transposition mechanism. Those transposing *via* an RNA intermediate belong to class I and are referred to as retrotransposons, whereas those transposing *via* a DNA intermediate belong to class II and are called DNA transposons [4]. Class I transposable elements can be classified into three main orders, LTR retrotransposons (having long terminal repeats), LINEs (long interspersed nuclear elements) and SINEs (short interspersed nuclear elements), whereas class II transposable elements are classified into DNA transposons, Helitrons and Mavericks [5]. Most TEs encode proteins that mediate their autonomous transposition. During the course of evolution, non autonomous elements have emerged from autonomous elements. Some are incomplete versions of autonomous elements, often with insertions/deletions (indels) disrupting their open reading frames (ORFs). Others are miniature versions lacking internal sequences but retaining the boundaries of the original element, making it possible for the autonomous element machinery to recognize them. MITEs (miniature inverted-repeats transposable elements) are well known examples of non autonomous elements that evolved from class II DNA transposons [6], [7].

TEs are now recognized to be a major component of the structure of the genome and to affect genome size and chromosomal rearrangements [8]–[10]. They often account for a large proportion of the genome: 20% of the 180 Mb *Drosophila melanogaster* genome, 45% of the 3.2 Gb human genome, and more than 80% of the 17 Gb bread wheat (*Triticum aestivum*) genome [11]. These dispersed repeats can induce major chromosomal rearrangements, thereby affecting genome organization. However, the impact of TEs is not limited to effects on genome structure. As initially suggested by Barbara McClintock [12], TEs may be seen as “controlling” elements. They may provide regulatory sequences with various effects on the adjacent genes. In particular, some silencing mechanisms involving RNA interference seem to have emerged primarily as a host response to prevent TE amplification. Thus, genes located close to TE insertions may be subject to transcriptional control due to TE repression, resulting in their epigenetic regulation [13], [14]. Moreover, TEs are intrinsically able to create, modify and re-wire gene regulatory networks [15], [16]. Finally, many cases of exaptation and domestication involving TEs have been reported [17], [18]. For example, there are several lines of evidence to suggest that the RAG1 and RAG2 genes involved in V(D)J recombination originated from a hAT DNA transposon that was domesticated to fulfill this primordial function of the adaptive immune system [19], [20].

The increase in efficiency and decrease in cost of new sequencing techniques [21] are leading to the sequencing of increasing numbers of genomes. About 1250 genome sequencing projects have already been initiated for eukaryotes, including species with large and repetitive genomes, such as maize [22]. The efficient and accurate annotation of TEs is therefore essential to our understanding of their impact on gene function and genome evolution [23].

If a routine TE annotation procedure is to be efficient, it must be both rapid and exhaustive, biologically relevant and computationally tractable. The TE annotation process can be divided into two phases: (i) the *de novo* discovery and identification of the TE families present in the genome studied and (ii) the precise, comprehensive annotation of TE copies on the chromosomes. For the second phase, an integrated pipeline has already been developed and tested [24] and this pipeline has been applied to several organisms [25]–[31]. For the *de novo* discovery phase, several programs and algorithms based on different assumptions have been developed, but none has yet proved entirely satisfactory. Indeed, as pointed out in a previous study [32], some programs have very low levels of sensitivity or specificity, whereas others return too short consensus sequence (<1 kb).

In addition to describing the composition and organization of the genome, TE annotation facilitates the identification of structural variants providing useful information about genome dynamics. Several examples of structural variations in TE families have been reported [33], [34] but these variations have generally been underestimated in genome-wide analyses of TEs. In this study, we addressed two questions, one concerning the challenges associated with whole-genome TE annotation, and the other relating to the identification and characterization of structural variants from the same TE family. We first compared the existing computational methods for the *de novo* identification of TEs in sequenced genomes, using the high-quality TE annotations available for the *Drosophila melanogaster* [24] and *Arabidopsis thaliana* genome sequences [29]. We then developed the TEdenovo pipeline, a tool combining several different programs, including procedures for the clustering of interspersed repeats, into a single framework, the REPET package (http://urgi.versailles.inra.fr/index.php/repet). Finally, by analyzing the *D. melanogaster* and *A. thaliana* genomes with the TEdenovo pipeline, we were able to obtain new insight into TE dynamics, highlighting structural variations emerging during the diversification of TE families and identifying putative new TEs absent from reference databanks.

## Results

### Comparative analysis of *de novo* approaches

We developed a three-step approach for comparing the efficiency of *de novo* TE detection methods (see [35] for a review), to provide a robust tool for identifying TEs in eukaryotic genomes: (i) the self-alignment of the input genomic sequences, (ii) the clustering of the resulting pairwise alignments, and (iii) the construction of a multiple alignment for each cluster from which a consensus sequence is derived (Figure S1). This process generates a databank of *de novo* consensus sequences representing putative TE families present in the genome analyzed, which can be used for the annotation of individual TE copies. We applied this three-step approach to the *D. melanogaster* release 4 and *A. thaliana* release 9 genome sequences. At each step, we evaluated several programs, comparing the efficiency with which they identified TEs with the aid of the high-quality TE sequence databanks (from the Berkeley *Drosophila* Genome Project and Repbase Update) and annotations available for these two reference genomes [24], [29].

Traditionally, the quality of *de novo* consensus sequences — the extent to which they correspond to full TE reference sequences rather than truncated versions — is not assessed. Validation is instead indirect: researchers annotate a genome sequence with RepeatMasker, using Repbase Update as TE databank, and consider the resulting TE annotations as the references. They then annotate the same genome with RepeatMasker, using the *de novo* consensus as TE databank, and consider these TE annotations as predictions. These two sets of annotations are then compared, by calculating sensitivity and specificity at the nucleotide level. The criterion used to estimate the quality of the *de novo* method is therefore the extent to which *de novo* predictions and reference annotations overlap. However, as we are particularly interested in TE dynamics, we need to assess the quality of the *de novo* library itself, by evaluating the extent to which full ancestral TE reference sequences are recovered. Such sequences, which originate from the reconstruction of a given element from its copies, are not only useful for subsequent TE annotation, but also provide a condensed view of the TEs in the genome. One way of assessing the quality of the *de novo* consensus sequences obtained with our three-step approach would be to compare these sequences with reference sequences from the Berkeley Drosophila Genome Project (BDGP) or Repbase Update databanks. However, it was clear that some of the reference sequences present in these databanks would not be present in the genomes analyzed. For example, the “P-element” reference sequence is absent from the genome sequence of the *D. melanogaster* strain used here. So, rather than using the reference databanks directly, we first constructed, for each genome, a “knowledge-based” databank comprising one consensus sequence per reference TE sequence, based on its genomic copies (see Methods). For each genome, we then compared each *de novo* databank with its corresponding “knowledge-based” databank through pairwise sequence alignments. We then calculated the sensitivity S_n_*, specificity S_p_* and recovery ratio R_CC_ (see Methods and Table 1). This last index, the R_CC_ ratio, provides a precise measurement of the number of TE reference elements fully recovered in the *de novo* consensus sequences.

**Table 1:** Sensitivity and specificity of the programs tested in the three-step *de novo* approach.

Genome	Self-alignment	Clustering	Multiple alignment	S_n_*	S_p_*	R_CC_
*D. mel.*	BLASTER	GROUPER	MAP	80.34%	85.89%	66.20%
*D. mel.*	BLASTER	RECON	MAP	92.31%	73.17%	66.20%
*D. mel.*	BLASTER	PILER	MAP	62.39%	84.17%	51.50%
*D. mel.*	PALS	GROUPER	MAP	73.50%	88.75%	60.30%
*D. mel.*	PALS	RECON	MAP	90.60%	74.23%	51.50%
*D. mel.*	PALS	PILER	MAP	53.85%	76.42%	42.64%
*A. tha.*	BLASTER	GROUPER	MAP	60.33%	82.42%	39.00%
*A. tha.*	BLASTER	RECON	MAP	73.77%	61.70%	43.50%
*A. tha.*	BLASTER	PILER	MAP	47.21%	57.33%	32.45%
*A. tha.*	PALS	GROUPER	MAP	54.75%	88.38%	24.00%
*A. tha.*	PALS	RECON	MAP	71.80%	66.20%	27.90%
*A. tha.*	PALS	PILER	MAP	40.00%	59.92%	16.20%

“*D. mel.*” stands for “*D. melanogaster*” and “*A. tha.*” stands for “*A. thaliana*”. The three indices S_n_*, S_p_* and R_CC_ correspond respectively to the measure of sensitivity, the measure of specificity and the recovery ratio when comparing a databank of TE *de novo* consensus sequences with a databank of TE reference sequences.

#### Self-alignment of the genomic sequences

The first step in the three-step *de novo* approach involves the self-alignment of the input genomic sequences, corresponding to an all-by-all comparison of the genome with itself. We evaluated two local pairwise alignment programs for this first step: a heuristic algorithm, BLASTER [36] wrapping BLAST [37], and an exact algorithm, PALS [38] (see Methods). We launched these two programs, with stringent parameters, on each of the target genomes, and then applied post-processing procedures to discard long segmental duplications (see Methods). This resulted in a list of pairwise alignments corresponding to repeats in the *D. melanogaster* and *A. thaliana* genome sequences. A comparison of the *de novo* consensus sequence performances of the BLASTER and PALS programs (Table 1) showed that BLASTER consistently had a higher sensitivity (S_n_*) and a much higher recovery ratio (R_CC_). In most cases, its specificity (S_p_*) was also better than that of PALS. Note that PALS was run with more stringent parameters than BLASTER as it cannot be used with the same values without computing time becoming intractable (see Methods). As the recovery ratio R_CC_ reflects the ability of the *de novo* approach to define TE boundaries correctly and, therefore, to recover full-length TE reference sequences, we preferred BLASTER over PALS for the self-alignment step. Moreover, in both genomes, BLASTER gave a higher genome coverage than PALS (Table S1), as expected given the lower stringency of its parameters (see Methods). This first step also generated a lower limit for the repeats content of the genome: around 7% for *D. melanogaster* and 13% for *A. thaliana*.

#### Clustering of the all-by-all matches

**Figure 1:**
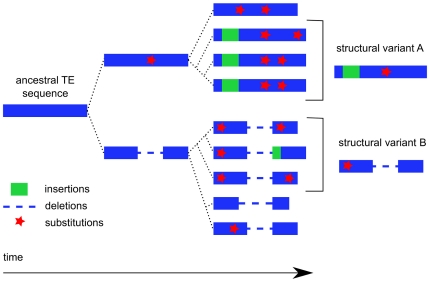
Schematic diagram of the dynamics of a TE family with two structural variants.

In the second step, we clustered the results of the all-by-all comparisons, with the aim of gathering together, within the same cluster, all sequences belonging to the same TE family. This step is crucial to ensure the precise definition of repeat boundaries, one of the main challenges in the *de novo* detection of TEs. Due to their specific dynamics during genome evolution, TE families may differ considerably in terms of copy number, sequence divergence and insertion/deletion patterns. At this step, the aim is to cluster together all TE fragments sharing a common ancestor, with the aim of recovering the ancestral element that transposed in the past. However, TE copies diverge as they multiply and different copies may accumulate different modifications. This phenomenon results in structural variants, as depicted in Figure 1. In this case, the grouping together of copies from different structural variants within the same cluster may have the undesirable consequence of the identification of consensus sequences with some features specific to one of the structural variants with others specific to another variant. We therefore tested three programs specifically implemented for the clustering of interspersed repeats: GROUPER [36], RECON [39] and PILER [40] (see Methods).

We applied these three programs to the list of matches obtained in the genome self-alignments, and then applied procedures for discarding segmental duplications (see Methods). In terms of the *de novo* consensuses generated from the resulting clusters (Table 1), GROUPER and RECON were consistently more sensitive than PILER. Moreover, GROUPER was systematically more specific than RECON and PILER, with *de novo* consensuses from GROUPER less likely to be artifacts unrelated to TE reference sequences than those obtained with the other methods. In some cases, RECON provided a better recovery ratio (R_CC_) than GROUPER and PILER, but this ratio was always better for GROUPER than for PILER. In addition, regardless of the genome analyzed, the three programs did not necessarily generate the same number of clusters (Table S2): GROUPER generated more clusters than RECON, which, in turn, generated more clusters than PILER. The number of clusters obtained by the *de novo* approach was much higher for GROUPER and RECON than the number of TE families present in the reference databanks (BDGP or Repbase Update). We suggest that this large number of clusters reflects the high level of diversity within TE families in terms of copy divergence and nesting patterns, as assessed by clustering algorithms.

#### Multiple sequence alignment for each cluster

The third step, which consists in the construction of a multiple alignment for each cluster, is particularly important, because the quality of a consensus sequence depends on the quality of the multiple sequence alignment (MSA) from which it is derived. A large number of programs have been developed over the last ten years [41], focusing on issues such as the scoring scheme, the use of templates for secondary structures and computation speed. In our case, the sequences to be aligned are very similar to each other in terms of nucleotide substitutions (identity of more than 90%), with most of the differences between them resulting from indels. The clusters generated by RECON include sequences of very different lengths (Table S2). Moreover, depending on the size and TE content of the genome analyzed, it may be necessary to process several thousand clusters. We tested only a subset of all the possible programs, focusing on those most suitable for our requirements: rapid procedures capable of handling indels. We therefore focused on progressive MSA algorithms, as these algorithms generate alignments rapidly. We tested MAP [42], CLUSTAL-W [43], MAFFT [44] and PRANK [45] (see Methods).

For each of these multiple alignment programs, we used the matches returned by BLASTER and then clustered by GROUPER, RECON or PILER. This comparative study (Table S3) shows that all the multiple alignment programs gave similar results in terms of sensitivity and specificity, whether launched after GROUPER or after PILER. This result was expected, as all the sequences in these clusters are similar in both composition and length, making them easy to align. However, PRANK was slower than the other three programs, rendering it less suitable for large genome analyses. With RECON, the best results were obtained with MAP, due to the greater heterogeneity of RECON clusters in terms of sequence length, rendering alignment more difficult. Thus, few differences were observed, but MAP clearly outperformed the other programs on RECON clusters, making it more robust than the other programs, whatever the clustering method used.

#### Comparison with another approach, RepeatScout

We evaluated the performance of our three-step strategy, by comparing it with another approach that also builds a databank of TE consensus sequences from a raw genome sequence: RepeatScout [46] (see Methods). We applied the RepeatScout program on the *D. melanogaster* and *A. thaliana* genome sequences, with the default parameters. It constructed 1770 consensuses for *D. melanogaster* and 3417 for *A. thaliana*. However, these consensuses were less sensitive and specific than those obtained with the tools described above (Table S4). This is probably due to the shorter length of the consensuses identified by RepeatScout (500 bp on average) than by GROUPER (∼2500 bp on average), RECON (∼2000 bp on average) and PILER (∼2800 bp on average). We obtained similar results for both genomes, indicating that this bias does not seem to be due to the input genome sequences. We hypothesize that RepeatScout fails to connect TE fragments more than a certain distance apart, thereby sometimes missing the true boundaries of a given TE copy. By contrast, GROUPER and RECON are particularly efficient at this task.

#### Combination of programs into a robust pipeline, TEdenovo

**Figure 2:**
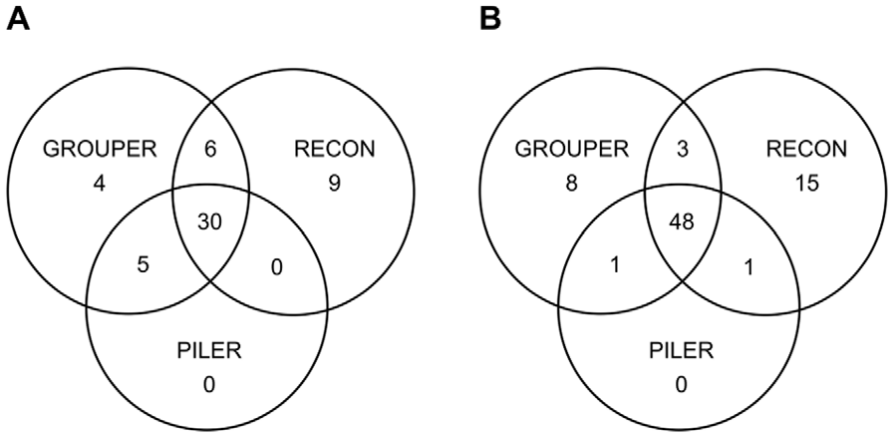
Venn diagram showing the gains achieved by combining several clustering programs. (A) Combining the GROUPER and RECON programs in particular makes it possible to fully recover more TE sequences than each program alone from the *D. melanogaster* genome. (B) Same conclusion from the *A. thaliana* genome.

Based on the comparative analyses reported above, BLASTER should be used for the genome self-alignment step, followed by GROUPER or RECON for the clustering step and MAP for the multiple alignment step. However, a comparison of the consensuses obtained with all three clustering methods clearly showed that each of these methods nonetheless missed several reference TEs fully recovered by the others (Figure 2). With the *D. melanogaster* genome sequence, four “knowledge-based” consensuses were retrieved intact by GROUPER only, and nine such sequences were recovered by RECON only (Figure 2 A). Similarly, with the *A. thaliana* genome sequence, eight “knowledge-based” consensuses were retrieved intact only by GROUPER, whereas fifteen were retrieved intact only by RECON (Figure 2 B). The three clustering methods should therefore be used in combination, for the accurate identification of TE families in genome sequences. For this reason, we decided to the three-step approach within a combined, modular pipeline named “TEdenovo”. With respect to the best single method, GROUPER for *D. melanogaster* and RECON for *A. thaliana* (Table 1), the combined approach, as implemented in the TEdenovo pipeline, improved the recovery of full-length “knowledge-based” consensus sequences by 20% and 13.5%, respectively, while maintaining high sensitivity and specificity (Table S5). The three approaches are combined at the clustering step, through the launching of GROUPER, RECON and PILER in parallel. The user may also choose to use PALS rather than BLASTER at step 1 or the other MSA programs at step 3, and can even choose to apply only one clustering program at step 2, although our results suggest that this would not be wise.

### Classification of TE consensus sequences and identification of structural variation within TE families

#### Classification of the consensus sequences and elimination of redundancy

**Figure 3:**
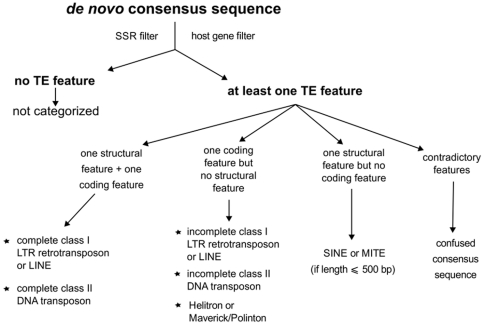
Simplified decision tree implemented in the TE classifier.

The three-step approach provided us with a set of *de novo* consensus sequences corresponding to interspersed sequences occurring at least three times in the genome studied. A two-step classification procedure was implemented to add more biological information, to filter out false-positives and to eliminate the redundancy introduced by the combined approach. There is a long-standing debate about the aims of any classification in biology [47], and the case of genomic repeats does not escape the rule: “Although the reality is that repeats […] are a hierarchical evolutionary continuum that defies classification, it is still desirable to impose a simplistic classification that pretends that repeat families are distinct, for the purpose of practical genome annotation” [39]. In this spirit, our classification procedure begins with the detection of TE features in the consensus sequences, and a decision tree classifying each consensus as a function of these features is then produced (Figure 3). In the first step, the procedure identifies terminal repeats, tandem repeats, poly-A tails and SSR-like tails (simple sequence repeats). It also aligns the consensus sequences with known TEs through blastn, blastx and tblastx, and with known genes from the host genome. The known TEs are those from Repbase Update, a curated set of TE sequences from numerous genomes [48]. Our TE classifier uses a customized version of this databank available from the Repbase Update website (http://www.girinst.org/repbase/), but any customized databank may be used, provided that it is appropriately formatted. In the second step, the procedure implements a decision tree based on the classification summarized in [5]. For benchmarking purposes, when analyzing *de novo* consensus sequences from the *D. melanogaster* and *A. thaliana* genomes, we removed the sequences known to belong to these species from the Repbase Update databank, to establish conditions equivalent to those for the analysis of a new genome.

The classification takes into account the degree of completeness of the *de novo* TE consensus (Figure 3). For instance, if a consensus sequence has the required “structural features” — LTRs (long terminal repeats), TIRs (terminal inverted repeats) or a tail (poly-A or SSR-like) — and “coding features” — matches with known TEs in tblastx and blastx analyses — then it is considered “complete”. If it has only one of these two types of features, it is classified as “incomplete”. Moreover, a consensus sequence can be classified as “confused” if our TE classifier detects features known to belong to different categories of TEs or, based on the detected features, its length is outside the range of TEs known to have such features. If the consensus has no identifiable features, it is classified as “not categorized”. We also used length parameters (Table S6) benchmarked on the reference databanks — BDGP for *D. melanogaster* and Repbase Update for *A. thaliana* — to improve differentiation between presumably truncated and full-length consensus sequences. We also used length parameters when classifying a sequence having only “structural features”, to determine whether the sequence concerned was a SINE or a MITE.

**Figure 4:**
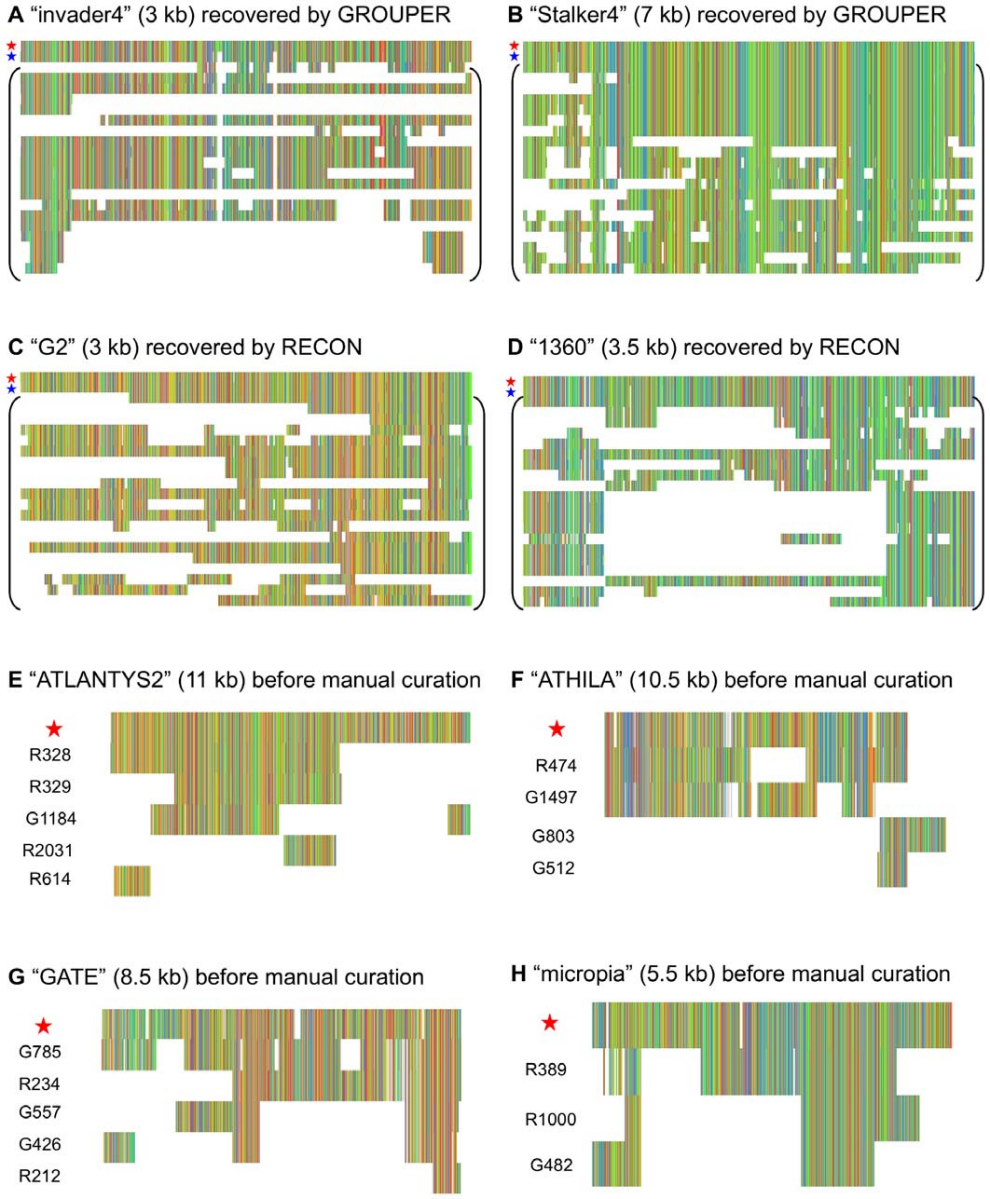
Extensive structural variations within several TE families. Each image provides an overview of a multiple alignment, a column being in one color if all the residues within it are identical. In all the images, the first sequence in the multiple alignment (red star) is the TE reference sequence from a public databank (BDGP or Repbase). For alignments A to D, the second sequence (blue star) is the only *de novo* consensus in which the TE reference sequence is fully recovered by only one clustering method. All sequences below (in brackets) are TE genomic copies found by the *de novo* consensus analysis. For alignments E to H, the sequences below the TE reference sequence are *de novo* consensus that require manual curation. Beside is indicated the program that build them, “R” for RECON and “G” for GROUPER.

As shown in Table 2 for *D. melanogaster* (Table S7 for *A. thaliana*), our TE classifier retrieved the appropriate classification for the reference TEs, thus predicting a correct classification for *de novo* consensus sequences. Moreover, each high-level category of the TE classification (*e.g.* “class I LTR-retrotransposon”, “class I LINE”, and “class II DNA transposon”) contained similar proportions of *de novo* consensus sequences and TE reference sequences from the BDGP or Repbase Update databanks. Thus, our procedure efficiently classified *de novo* consensus sequences into the appropriate high-level categories.

**Table 2:** TEclassifier results for the classification of *D. melanogaster* TE sequences.

Classification	Reference TEs from the BDGP	*De novo* consensus (with redundancy)	*De novo* consensus (without redundancy)
Class I “complete” LTR retrotransposon	56	150	48
Class I “incomplete” LTR retrotransposon	2	377	209
Class I “complete” LINE	23	117	27
Class I “incomplete” LINE	17	147	57
Class I SINE	0	2	1
Class II “complete” DNA transposon	19	30	13
Class II “incomplete” DNA transposon	2	75	32
Class II MITE	0	8	5
Helitron	1	0	0
SSR	0	8	8
Host genes	0	26	11
Confused	1	20	6
No category	5	341	176
Total	126	1301	593

The proportion of *de novo* consensus sequences classified as “incomplete” was high. In *D. melanogaster*, 75% (45% in *A. thaliana*) of the *de novo* consensus sequences classified as “incomplete” LTR retrotransposons matched only with known LTR retrotransposons from Repbase Update but contained no long terminal repeats. All the *de novo* consensus sequences classified as “incomplete” LINE retrotransposons from *D. melanogaster* (28% of those from *A. thaliana*) matched only known LINE retrotransposons from Repbase Update, but with no polyA-/SSR-like tail. Among the *de novo* consensus sequences classified as “incomplete” DNA transposons, 50% of those from *D. melanogaster* (32% of those from *A. thaliana*) matched only known DNA transposons from Repbase Update, but with no TIR. Thus, these *de novo* consensus sequences are classified as “incomplete” in *D. melanogaster* mostly because they lack terminal features, which is not the case for most such consensus sequences in *A. thaliana*. Thus, the clustering programs combined in the TEdenovo pipeline do not generate systematic bias towards “incomplete” TEs of a given kind.

Moreover, only a few consensus sequences were classified as “confused”, facilitating manual curation. For instance, one 635-bp long consensus sequence built by RECON from the *D. melanogaster* genome has long terminal repeats as well as a poly-A tail which is contradictory according to our classification scheme derived from [5]. The consensus sequences classified as “host genes” are detailed in Table S8. The high proportion of unclassified consensus sequences (“no category”) reflects the limitations of this classification scheme. We searched all these consensus sequences for HMM profiles specific to TEs. We detected fragments of known TE profiles in only 14% of these unclassified consensus sequences (data not shown). Several of the other corresponded to Helitron reference sequences recovered intact within unclassified *de novo* consensus sequences. Helitrons are difficult to detect as they have no clear structural features other than a terminal hairpin [49]. Nonetheless, our *de novo* approach recovered all those with at least three full-length copies in the genome. This confirms the relevance of the combined *de novo* approach as implemented in the TEdenovo pipeline and shows that *de novo* consensus sequences may correspond to TEs even if they remain unclassified by this method.

As shown above, the combination of clustering programs gave better results than any single program. However, it also provided redundant consensus sequences. We therefore applied a redundancy elimination procedure, in which we considered a consensus sequence to be redundant if it was included in another sequence, over *x*% of its length, with an identity of more than *y*%. We first tested this procedure directly on the whole *de novo* databank. It resulted in the loss of many well classified consensus sequences that were shorter than the misclassified sequences. We therefore applied the redundancy procedure on the basis of the classification. We decided to remove redundant consensus sequences classified as “incomplete” when they were included within consensus sequences classified as “complete”, but not vice versa. The “80-80-80” rule [5] has been proposed as a means of identifying copies from the same TE family: two TE copies may be considered to belong to the same family if they are aligned, with 80% identity, over at least 80 bp and 80% of their respective lengths. However, as this rule was originally developed for TE copies and not for consensus sequences, we also tested more stringent parameters (Table S9). We found that the best strategy for obtaining a high-quality *de novo* databank with a low level of redundancy was to remove redundant consensus sequences with more stringent parameters: “95-80-98”. This implies that a consensus sequence is removed if it is included within another consensus sequence over 98% of its length, with an identity level exceeding 95%.

#### Comparison of de novo and knowledge-based TE annotations

After the first phase of analysis with the TEdenovo pipeline, we used the *de novo* TE consensus sequence databank to detect all TE fragments and to reconstruct each TE copy in the genome of interest. This annotation phase was achieved with the TEannot pipeline [24] (Figure S2), which is also part of the REPET package. TEannot combines several programs for detecting TE fragments, filtering out false-positives and reconstructing intact TE copies. This process involves the connection of TE fragments from the same copy, a procedure also called “TE defragmentation”. While improving the robustness of this pipeline, we notably improved the connection of TE fragments in the MATCHER algorithm (see Methods). For a comprehensive analysis of the performance of the *de novo* approach presented above, we used the TEannot pipeline on both the *D. melanogaster* and *A. thaliana* genomes, with several databanks of *de novo* consensus sequences, each obtained with a specific combination of programs from the TEdenovo pipeline. We first discarded the consensus sequences that could be unambiguously identified as SSRs or host genes. We then compared each annotation with that obtained with the reference TE libraries from the BDGP and Repbase Update databanks (Table 3).

**Table 3:** TE annotation results obtained with reference databanks and *de novo* databanks.

Genome	TE databank	Consensus (having copies)	TE genome coverage	Number of copies	S_n_	S_p_
*D. mel.*	BDGP	125	10.51%	31208	NA	NA
*D. mel.*	GROUPER	712	10.29%	43699	81.92%	98.12%
*D. mel.*	RECON	437	11.05%	33072	87.77%	97.95%
*D. mel.*	PILER	114	8.87%	32789	74.07%	98.79%
*D. mel.*	RepeatScout	1432	10.86%	42048	85.28%	97.88%
*D. mel.*	G+R+P	568	11.98%	42847	91.43%	97.35%
*A. tha.*	Repbase	318	19.02%	41146	NA	NA
*A. tha.*	GROUPER	1237	18.78%	41791	79.29%	95.43%
*A. tha.*	RECON	1004	23.69%	49470	88.75%	91.59%
*A. tha.*	PILER	300	13.14%	34818	56.56%	97.05%
*A. tha.*	RepeatScout	2893	21.95%	68958	82.91%	92.36%
*A. tha.*	G+R+P	1232	22.77%	44059	87.03%	92.32%

“*D. mel.*” stands for “*D. melanogaster*” and “*A. tha.*” stands for “*A. thaliana*”. The S_n_ and S_p_ columns correspond respectively to sensitivity and specificity results when comparing two annotations in terms of nucleotide overlaps. “G+R+P” indicates that the three programs GROUPER, RECON and PILER were used to build the databank of *de novo* consensus sequences.

In *D. melanogaster* (Table 3), when using only one clustering method, the GROUPER databank delivered the annotation closest to that obtained with the BDGP reference databank in terms of genome coverage and copy number. Sensitivity was highest with the RECON databank and specificity was highest with the PILER databank. The annotation shows high sensitivity and specificity, together with a high level of genome coverage, for the combined approach. Similar conclusions were drawn from the annotations for *A. thaliana*. An examination of match boundaries (Figure S3 and Table S10) showed that the clustering methods were complementary: GROUPER gave the largest number of exact matches in *D. melanogaster* whereas RECON gave the largest number of exact matches in *A. thaliana*. About RepeatScout, although the specificity of its annotation is very similar with our combined approach, its sensitivity is much lower. Moreover, based only on Table 3, it may appear that using “RECON” gives similar results than using our combined “G+R+P” approach. However, as shown above in Figure 2, “RECON” is not able to fully recover several TE reference sequences which are fully recovered only by “GROUPER”, and reciprocally. Biologically speaking, it is better to recover a full TE reference sequence as one consensus sequence, rather than several truncated or artifactual consensus sequences. In this context, not only the “G+R+P” approach builds a better library of TE *de novo* consensus sequences, but such a library is also able to provide reliable TE annotation. Thus, the combination of clustering methods in the TEdenovo pipeline leads to the construction of a high-quality TE library delivering annotations similar to those obtained for manually curated databanks.

We then compared the results of our analyses with those obtained with RepeatModeler (http://www.repeatmasker.org/RepeatModeler.html), which combines RECON, RepeatScout, RepeatMasker and TRF and classifies the consensus sequences obtained. For *D. melanogaster*, RepeatModeler generated a library of 141 consensus sequences, with a sensitivity of 78% and a specificity of 76%. However, the recovery ratio of RepeatModeler (R_CC_ = 21%) was much lower than that of the TEdenovo pipeline (R_CC_ = 72%). This indicates that RepeatModeler recovers only a few intact TE reference sequences. Similar results were obtained for *A. thaliana*, with all three measurements showing lower values with RepeatModeler than with TEdenovo (Table S11). Running the TEannot pipeline with the consensus sequences generated by RepeatModeler resulted in a sensitivity markedly lower than that for the annotations obtained with the *de novo* library from the TEdenovo pipeline, although specificity was slightly higher (Table S12). The combination of several tools is therefore not sufficient in itself to improve the results. The way in which the tools are chosen and combined is determinant. We conclude that the TEdenovo pipeline achieves a good balance between sensitivity and specificity in the *de novo* construction of a TE databank from raw genomic sequences.

We therefore developed the REPET package available online (http://urgi.versailles.inra.fr/index.php/repet), into which we integrated both the TEdenovo and TEannot pipelines, with the TE classifier described above corresponding to the final step of the TEdenovo pipeline. The REPET package was specifically designed to improve speed and tractability by (i) interacting with MySQL tables at several key points to take advantage of the SQL language, and (ii) automatically handling jobs launched in parallel on a cluster *via* free batch-queuing systems, such as the Sun Grid Engine (now known as Oracle Gene Engine), relaunching jobs in cases of cluster node failure. This package is thus specifically implemented for computationally intensive, genome-wide analyses that do not compromise the biological relevance of the results (see Table S13 for details about computation times). As already pointed out by several authors, tools for *de novo* TE identification are “quite difficult to use, indicating the need for better user interfaces and auto-optimization” [32]. We facilitated the use of our tools, by concealing technical details behind interfaces, one per pipeline. The user is also provided with access to a detailed tutorial and a configuration file with default parameters.

#### Identification of structural variation within TE families and manual curation

After annotating the TE copies in both genomes, we investigated the structural diversity within TE families, as represented by the large number of *de novo* consensus. We focused on the TE families for which the “knowledge-based” consensus was fully recovered by only one clustering method, GROUPER or RECON, as shown in Figure 2 (see also Table S14 and Table S15). Indeed, the failure of one method (either GROUPER or RECON) to recover all the TEs would illustrate differences in the ability of these methods to take TE structural variations into account. For each of these TE families, we retrieved genomic copies detected by the *de novo* consensus and built multiple alignments (see Methods). Figure 4 (cases a, b, c and d) provides an overview of several of these multiple alignments displaying extensive structural variations. In almost all TE families, differences between genomic copies were observed, due to substitutions and indels. The clustering method generated different clusters as a function of these differences and the fragmentation of the copies. Depending on the specific features of each algorithm, the consensus will correspond to the complete TE reference sequence or a truncated version of that sequence.

We then looked for features particular to the TE families for which the reference sequence was fully recovered by only one clustering method, being recovered only partially with another method. We first compared the classification of these TE reference sequences. In *D. melanogaster*, the four TE reference sequences fully recovered only by GROUPER were all LTR retrotransposons, whereas the nine TE reference sequences fully recovered only by RECON comprised four LTR retrotransposons, three LINE retrotransposons and two DNA transposons. In *A. thaliana*, the eight TE reference sequences fully recovered only by GROUPER comprised five LTR retrotransposons and three DNA transposons, whereas the fifteen TE reference sequences fully recovered only by RECON comprised six LTR retrotransposons, eight DNA transposons and one Helitron. There were therefore no clear differences in the recovery of full-length TE reference sequences obtained with different clustering methods. We also looked for differences in terms of copy number. However, the TE reference sequences fully recovered only by GROUPER or only by RECON had similar numbers of full-length and truncated copies (data not shown). Comparisons of *de novo* consensuses and “knowledge-based” consensuses, as in Table 1, showed that RECON was systematically the most sensitive method, whereas GROUPER was systematically the most specific. As a result, several TE reference sequences displayed partial matches with *de novo* consensuses from RECON only. Moreover, almost 50% of the *de novo* consensus sequences from RECON lacked both boundaries of the TE reference sequences, versus less than 20% of the consensus sequences from GROUPER. Conversely, a *de novo* library from GROUPER is likely to match with fewer TE reference sequences, but, when a match does occur, at least one boundary of the reference is likely to be correctly retrieved. Consequently, GROUPER and RECON are truly complementary, making the combined approach implemented in the TEdenovo pipeline very efficient.

Our TE classifier can combine *de novo* consensus sequences from various sources, but manual curation is still required in some cases, particularly when there are several consensus sequences related to the same TE family (Table S16). Figure 4 (alignments E to H) shows several examples in which manual curation of the *de novo* consensus sequences identified by GROUPER and RECON for a given TE family improves the recovery of the reference sequence. For instance, in Figure 4-E, all consensus sequences from RECON are truncated before the 3′ LTR of the ATLANTYS2 element whereas the G1184 consensus sequence from GROUPER connects this LTR with part of the internal region of the element. In Figure 4-F, two consensuses can be removed, G512 from GROUPER, a a solo-LTR, and G803, which is chimeric. Moreover, we can add the internal region of the element present in G1497, which lacks the 3′ LTR, to the truncated R474 from RECON. A similar strategy can be applied in figures 4-G and 4-H. Thus, although it is not always possible to recover the full reference sequence, we can improve the final *de novo* consensus by manual curation, making use of the multiple alignment of classified consensuses to guide informed decision-making.

The TEdenovo pipeline identified putative new TEs in the *D. melanogaster* and *A. thaliana* genomes, despite the intensity with which these genomes have been studied and annotated manually. Indeed, we found *de novo* consensus sequences that were classified as “complete” TE but had no single match in blastn with known TE reference sequences from the BDGP or Repbase Update databanks. Three such sequences were identified in *D. melanogaster* (two from GROUPER and one from RECON), and four in *A. thaliana* (three from RECON and one from PILER). For example, in *D. melanogaster*, a 7.8 kb *de novo* consensus was classified as a “complete” LTR retrotransposon on the basis of its two long terminal repeats (each 510 nt long) and its matches with known LTR retrotransposons from Repbase Update in tblastx and blastx analyses. Furthermore, this consensus matched two HMM profiles corresponding to an integrase and an aspartic proteinase. There were four full-length copies in the genome, two of which retained their target site duplications. This *de novo* consensus sequence is probably non autonomous, as it lacks matches with HMM profiles corresponding to other LTR retrotransposon genes, and, notably, displays no match with a reverse transcriptase. However, it has enough of the typical properties of TEs and enough full-length copies to correspond to a true TE, rather than a mere segmental duplication containing TE fragments.

## Discussion

### Combining approaches to build high-quality TE *de novo* consensus sequence databanks for sequenced genomes

Transposable elements play a key role in the structure and evolution of genomes, but their impact remains to be fully elucidated. If we are to make use of the increasing numbers of genome sequencing projects to improve our understanding of TE biology, we will need an efficient, automatic *de novo* approach for the annotation of genome TE content. Several methods praised for their rapidity and low memory requirements, such as the MDR index [50], and P-clouds [51], provide a good overview of the repeat content of a given genome. However, they are not precise enough to provide insight into the functional impact of TEs (see for instance [52] and [53]). As such, these methods are good starting points but are inadequate for a full exploration of biological questions relating to TEs.

The typical process of TE annotation, as we see it, should involve the identification of all TE copies throughout the genome and proceed in two phases. The first step is the construction of a *de novo* library of TE consensus sequences representing the TE families present in the genome. This library is then used to mine all the TE fragments present in the genome. The TE copies are then reconstructed, usually nested within each other, to unravel their intricate evolution. We describe here a new strategy for constructing databanks of TE consensus sequences by *de novo* methods. Our results for the *D. melanogaster* and *A. thaliana* genomes show that an approach combining several clustering programs provides the best outcome, in terms of sensitivity and specificity, for the identification of TE families and the annotation of their TE copies.

Previous studies have already compared tools for *de novo* TE identification, either to propose a new algorithm (as for RECON, PILER and RepeatScout) or to help experimental biologists to use the most suitable tools for their analysis [32]. In most of these studies, the results were validated by comparing the final TE copy annotations in terms of genome coverage, rather than by evaluating the accuracy of *de novo* TE consensus sequence identification. Little attention has been paid to analyses of the *de novo* databank itself. The use of consensus sequences corresponding to truncated or artifactual TEs would clearly bias subsequent annotations. We therefore propose three indices for measuring the quality of *de novo* databanks, with respect to reference databanks. This combined approach was validated by considering the proportion of *de novo* consensus sequences corresponding to full-length reference sequences, with well-defined boundaries.

### Classification of TE sequences on the basis of their biological features and the curation of TE *de novo* databanks

The tools typically used for the *de novo* identification of TE families provide no additional meta-data about the consensus sequences they build. Instead, the user has to use other tools to obtain a putative classification of the sequences obtained. Here, we implemented a combined *de novo* approach for the identification of TE families and developed a dedicated procedure for classifying sequences on the basis of TE features. We based our TE classifier on the classification proposed in [5], from class to superfamily, combining various sources of information about TEs in a modular manner. This procedure was designed to be run in isolation for the analysis of any databank of putative TE-related sequences and for integration into the TEdenovo pipeline, to facilitate its use.

The current version of our tool, TEclassifier, requires further improvement for the classification of less well known TEs, such as DIRS-like elements, Penelope-like elements, Crypton elements and Polintons/Mavericks. However, unlike tools like TEclass [54], which makes use of the profiles of frequent *k*-mers from the Repbase Update databank, our TE classifier guides output classification by detailing the precise TE features present on the analyzed sequences, such as terminal repeats and matches with known TEs. Our tool and REPclass [55] are very similar, but our TE classifier has several advantages. Being part of the REPET package, it benefits from the architecture of this package and is therefore able to handle large databanks, which is often required when studying large genomes. Moreover, our TE classifier not only classifies putative TEs, it also makes it possible to filter out false-positives and to remove redundant sequences present in the input databank.

In a wider perspective, we considered manual curation, a key topic in genome annotation. Computer-based predictions for the annotations of protein-coding genes are now of high quality, but it remains difficult to predict exon-intron boundaries correctly [56]. Major efforts have been and are being made to improve the automatic annotation of protein-coding regions, but such efforts have been much less intensive for other parts of the genome, including TEs. In this study, we tried to fill this gap by designing the REPET package for the comprehensive association of all the meta-data obtained for a given TE family: (i) the *de novo* consensus sequences, (ii) the TE features used to classify them, (iii) the all-by-all comparisons from which they were built, and (iv) the TE copies they identify on the chromosomes. As a result, the manual curator can make informed decisions based on the biological features at hand, and the curated TE consensus databank can be used to provide a second release of TE annotations.

### Efficiency of *de novo* approaches for recovering ancestral TEs and their structural variants from “junk”

At the core of any *de novo* approach lies the possibility of identifying new agents in a given system by searching for their fundamental properties. In our case, we were confronted with raw whole-genome sequences containing numerous repeated sequences of different kinds, some of which have specific features and a common ancestry. We were interested in identifying, from among these sequences, TEs — interspersed repeats — and, more particularly, the ancestral sequences that actually transposed. The current copies of such sequences are likely to be divergent, fragmented and nested within each other, as TE families typically display extensive structural variation (Figure 4 A–D). We showed in this study that the *de novo* approach could recover some full-length TE sequences by correctly collecting together their fragments, even if no TE feature could be used to classify them. This was the case here for several Helitron reference sequences in the *A. thaliana* genome. Similarly, we were able to identify putative new non autonomous TEs in the *D. melanogaster* and *A. thaliana* genomes that had all the essential features of TEs but were nonetheless absent from the reference databanks.

Efficient and robust tools are essential to keep pace with current whole-genome sequencing programs. Our analyses were performed on small model genomes. However, the *de novo* approach and the tools we presented here are scalable, at least to genomes reaching 500 Mb. Indeed, preliminary versions of the TEdenovo and TEannot pipelines were used to annotate the TE content of the 464-Mb aphid genome totalizing 38% of TEs [31]. Moreover, our tools are modular and almost all steps can be launched in parallel, thus taking advantage of today's computer clusters. Future work will be dedicated to the improvement of the clustering step in the TEdenovo pipeline in order to provide, also to very large genomes, the ability to jointly annotate TEs and study their diversification. For all projects in which the aim is to sequence large and highly repetitive genomes (*e.g.* barley, hexaploid wheat), computational tools, such as the TEdenovo pipeline presented here, are likely to become increasingly useful for increasing our knowledge of the evolution of genome structure and the functional impact of TEs on neighboring genes.

## Methods

### Genome sequences and TE reference databanks as benchmark

In 2000, an international consortium driven by the Berkeley Drosophila Genome Project (BDGP) sequenced, assembled and annotated the genome of an isogenic *y*; *cn bw sp* strain of *D. melanogaster* [57], this work now being continued at FlyBase (http://flybase.org/). In this study, we used the 118.4 Mb release 4 genome sequence corresponding mostly to euchromatin regions. Release 5 became available in 2006 and corresponds to release 4 with 50.3 Mb of additional heterochromatic regions and minor sequence corrections. We chose to work on release 4, which remains the *D. melanogaster* genomic sequence best annotated for TEs [24], [25], despite the availability of the later sequence.

Flybase provides a set of natural TE sequences experimentally identified in several Drosophilidae genomes. The latest version of this data set (bdgp9.41) consists of 179 sequences corresponding to different TE families, 126 of which have been detected in *D. melanogaster* strains. Each of these 126 sequences corresponds either to a TE copy of the given family present in *D. melanogaster* or to a consensus based on TE copies for the family concerned. In the latter case, consensuses were built either as a mosaic of TE copies or following a “majority rule” from a multiple alignment. In this study, these 126 sequences are referred to as “reference TEs”. The aim of the *de novo* approach presented in this article is to reconstruct such a library of TE sequences.

In 2000, 115 Mb of the 125 Mb genome of *A. thaliana* accession Columbia had been sequenced, assembled and annotated [58]. For our analyses, we used the 119 Mb of *A. thaliana* release 9 genome sequences available from the TAIR website (http://www.arabidopsis.org/). For *A. thaliana*, a similar set of reference TEs is present in the Repbase Update databank. We used the library derived from this databank for previous studies [29] and containing 318 TE sequences.

We annotated both genomes with the TEannot pipeline [24] using the reference databanks cited above. We then constructed a consensus for each reference sequence, from the multiple alignment of copies when at least three copies longer than 100 bp were present (Table S17). For *D. melanogaster*, a consensus could be obtained for only 117 of the 126 reference sequences in the BDGP databank, referred to as a “knowledge-based” consensus: 68 of these sequences corresponded exactly to their reference element, the others being truncated. For *A. thaliana*, it was possible to construct 305 “knowledge-based” consensuses for the 318 TE reference sequences in the Repbase Update databank: 154 corresponded to the entire element and the others were truncated. These consensuses were collected into “knowledge-based” databanks. As such, they represent the upper limit of what can be retrieved with the *de novo* methods compared in this study (Table S18).

### *De novo* detection of TEs

A three-step approach forms the backbone of our analysis: (i) self-alignment of the genomic sequences, (ii) clustering of the resulting matches, and (iii) multiple alignments of each cluster to build a consensus (Figure S1). Different programs may be used at each step, leading to several possible combinations. For each combination, we compared the *de novo* consensus with the set of reference TEs. Other methods are also available for constructing a consensus for comparison with the “knowledge-based” databanks constructed as described above.

When comparing a *de novo* databank with a “knowledge-based” databank, it is not possible to calculate the number of false-negatives, *i.e.* the *de novo* consensus sequences incorrectly classified as not related to a TE because these consensus sequences are not present in the *de novo* library. The usual definitions of sensitivity and specificity are therefore not directly applicable. We estimated sensitivity by calculating the proportion of “knowledge-based” consensus sequences matching *de novo* consensus sequences (noted S_n_*). We then estimated specificity by considering the proportion of *de novo* consensus sequences matching “knowledge-based” consensus sequences (noted S_p_*). The observation of a *de novo* consensus that matches a “knowledge-based” consensus, both aligned along their entire lengths (+- 5%) indicates that this *de novo* consensus retrieved the exact and entire “knowledge-based” consensus. A recovery ratio (noted R_CC_ for “complete-complete ratio”) can be calculated as the number of “knowledge-based” consensus sequences exactly retrieved by a *de novo* consensus, divided by the number of reference sequences exactly retrieved by a “knowledge-based” consensus. In *D. melanogaster*, 68 reference sequences (of the 126 in the BDGP databank) were exactly retrieved by a “knowledge-based” consensus, and 154 such sequences (of the 318 in Repbase Update) were exactly retrieved in *A. thaliana*. As an illustration, in *D. melanogaster*, if a given *de novo* method recovers 60 entire “knowledge-based” consensus sequences from a given genome, R_CC_ would be 88% (60/68).

#### First step: self-alignment of the genomic sequences

The first step is the self-alignment of the genomic sequences in an all-by-all manner. We compared two programs, BLASTER [36] and PALS [38] for this purpose. BLASTER is a wrapper for the BLAST softwares [37]. It was used for comparisons at the genome scale. It begins by cutting long queries into batches and launching them in parallel against the subject databank. The second program, PALS, implements a filter algorithm. It first finds all exact matches of length *q* between the query and subject sequences. It then restricts the search by identifying regions (parallelograms in the alignment matrix) containing a number of hits above a given threshold. Finally, if these regions have a length above a given threshold, PALS develops a chain of hits for each region, aligns the nucleotides and returns the coordinates of the resulting matches. For BLASTER, we kept matches with E-value below 10^−300^, a length exceeding 100 bp and an identity exceeding 90% (parameters “-E 1e-300 -L 100 -I 90”). For PALS, we used the default parameters: identity exceeding 94% and a length of more than 400 bp (parameters “-length 400 -pctid 94”). If the same parameters were used for BLASTER (identity >90% and length >100 bp), the computations took too long, even when launched in parallel. If the parameters length >100 bp and identity >94% were used with both programs, BLASTER still gave better results (data not shown).

When all the matches had been retrieved, we discarded all those that were more than 20,000 bp long. This procedure was designed to filter out repeats corresponding to long segmental duplications. The issue of short segmental duplications was addressed in the next step. To speed up the computations, the input genome sequences were cut into chunks of length 200 kb with 10kb overlaps. These values are parameters of the tool and can thus be changed easily, according to the genome size, its repeat content, as well as technical details such as the number of nodes in the computer cluster. Each chunk was then aligned against the whole set of chunks, in parallel. Finally all the matches were collected together and redundant matches were filtered out.

#### Second step: clustering of the resulting pairwise alignments

The matches obtained in the first step were then clustered, and we tested three different programs for this step: GROUPER ([36], parameters “-j -C 0.95 -Z 3 -X 2 -G -1”), RECON ([39], default parameters) and PILER ([40], parameter “-trs”).

As TEs are frequently inserted within each other, GROUPER first aims at retrieving the full-length copies by connecting their fragments *via* a dynamic programming algorithm applied to the pairwise alignments. It links together, in chains, the matches corresponding to fragments belonging to the same TE copy but interrupted by indels. It then uses single-link clustering to gather similar chains of matches into the same cluster, using a high-coverage constraint (95%), and merges chains corresponding to the same locus. The use of a high-coverage constraint makes it possible to identify different structural variants from a given TE family (Figure 1): two copies from two different variants overlap by less than 95% threshold, and are therefore assigned to different clusters. The previous version of GROUPER suffered from the high redundancy among clusters, preventing its usage in practice on large genomes. Therefore, we implemented a new procedure that specifies, during the single-link clustering step, if a chain of matches is fully included within another one, as is often the case with non-autonomous TEs with respect to their autonomous counterparts. Once the clusters are built, we now remove those having less than a given number of members not included in any others (option “-X 2”).

RECON uses a different strategy, first trying to infer the ancestral TE copies, named “elements”, from all the fragments at each locus. It does this by single-link clustering with a low-coverage constraint (50%), followed by an additional procedure focusing on the aggregation of endpoints to ensure the correct handling of composite elements, such as segmental duplications. It then gathers the “elements” into families again by single-link clustering, but this time with a high-coverage constraint (90%), and a procedure is then applied to deal with families that are related but different, based on length ratio and similarity thresholds.

We also tested the PILER suite of programs. In this work, “PILER” refers systematically to PILER-DF, which focuses on interspersed repeats. PILER first defines “piles” as lists of matches covering a maximal contiguous region. It then globally aligns these piles, rather than the individual matches themselves. This prevents bias towards the shortest match in the pile. Finally, piles that cannot be globally aligned with each other over 95% of their length are gathered into clusters.

All these three programs, GROUPER, RECON and PILER, return a set of clusters to which we applied several filters. First, we removed the clusters with fewer than three members. This discarded most of the short segmental duplications. Second, for large clusters, we retained only the 20 longest sequences, as keeping all the sequences would not add much information for the building of a consensus and would even introduce noise (data not shown). Third, for GROUPER, we filtered the sequences resulting from connected matches (*i.e.* chains) with a cumulative length greater than 20 kb and spanning more than 30 kb of the genome, as such sequences probably corresponded to segmental duplications.

#### Third step: multiple alignments and consensus construction

Finally, for each cluster, a multiple sequence alignment (MSA) was constructed, from which a consensus was derived. We compared several programs: MAP ([42], parameters “gap-size = 50 mismatch = −8 gap-open = 16 gap-extend = 4”), CLUSTAL-W ([43], default parameters), MAFFT ([44], parameter “–auto”) and PRANK ([45], parameter “-F”). The consensus was constructed by applying a majority rule discarding columns in which all but one sequence had a gap.

MAP was specifically designed to handle long gaps. Such gaps frequently occur when aligning TE copies of different lengths from the same TE family. In this program, gaps are not penalized beyond a given length. CLUSTAL-W is a well known progressive MSA algorithm, and was the first to propose position-specific gap penalties. MAFFT addresses the issue of CPU time by using Fast Fourier Transform for the rapid detection of homologous segments. It also implements a normalized similarity matrix that is said to perform better for alignments with sequences of different lengths. This program was the fastest MSA program we tested. PRANK takes into account the phylogenetic information contained in indels to distinguish insertions from deletions, to position them properly and avoid the overestimation of deletions. This sophistication renders PRANK much slower than MAP (10 times slower for a typical cluster containing 16 sequences of 8 kb each).

#### Other approaches

Other methods have also been proposed. We tested REPEATSCOUT [46] and RepeatModeler (Smit and Hubley unpublished), both with default parameters. RepeatScout begins by keeping high-frequency strings of length *k*, called *k*-mers. The program initially takes the most frequent *k*-mer and sets the consensus as being the *k*-mer in question. Based on the multiple alignment of all its occurrences, the program extends this consensus one nucleotide at a time, in both directions, according to a specific scoring function. For correct definition of the consensus boundaries, the scoring function is designed to allow extension of the consensus, even if shared by some alignments, but not all. Once the consensus can be extended no further, the program detects all its occurrences in the genome, and updates the initial table of *k*-mer frequencies accordingly. This procedure is applied iteratively for each *k*-mer with a frequency above a given threshold.

### Classification of TE consensus and the elimination of redundancy

We implemented a two-step TE classifier. The first step detects structural features of the consensus, such as terminal repeats, tandem repeats, and polyA or SSR-like tails, using programs from the REPET package (TRsearch, polyAtail) or elsewhere (TRF, [59]). It also searches for matches with known TEs, by blastx, tblastx and blastn analysis, and for matches with known genes from the host's genome, by blastn analysis. The second step is based on a decision tree (Figure 3) classifying each consensus according to its length and features. The classification and the evidence underlying the classification are delivered as output. Any program looking for other TE features, such as Helitron hairpins, could easily be integrated into this framework.

We eliminated redundant consensus sequences, by discarding all those included within another consensus sequence, for at least *x*% of their length, and with at least *y*% identity. We tested several values for *x* and *y*: 95-98, 90-90 and 80-80. We tested this procedure with and without taking into account the classification of the sequences.

### Genome-wide annotation of TE copies

A combined pipeline named TEannot for the genome-wide annotation of TE copies is already available [24] (Figure S2). As input, it takes the genome sequences and a databank of TE sequences, typically that generated by the TEdenovo pipeline. It then launches BLASTER [36], RepeatMasker (http://www.repeatmasker.org/) and CENSOR [60], to map the TE sequences against the genome. False-positives are filtered out by applying the same procedure to shuffled genomic sequences. More precisely, the genomic sequences are randomized using the “shuffle” program of the HMMER package (http://hmmer.janelia.org/). The TE reference sequences are then mapped onto these shuffled sequences with BLASTER, RepeatMasker and CENSOR, with the score for each match recorded. Finally, the matches between the TE reference sequences and the true genomic sequences are filtered according to these scores. Whereas in a previous version of the pipeline we used the highest score obtained on randomized genomic sequences to filter false-positives, we now use the 95% quantile of the scores obtained on the randomized sequences. This improvement prevents excessive filtering, using a single, very good match on randomized sequences, much better than most others. This change slightly increases TE coverage over previous estimations.

Once the matches were filtered, we began to reconstruct the TE copies, to obtain a true annotation of TE copies and not of TE fragments only. In this pipeline, two steps are used to connect several TE fragments belonging to the same TE copy: MATCHER [36] and the “long join” procedure. As in the *de novo* library, a TE family may be represented by several consensus sequences corresponding to each of its structural variants. We improved these tools to take this into account. In terms of vocabulary, we define a “TE fragment” as a match between a TE consensus sequence and a genomic sequence, whereas we define a “TE copy” as a chain of matches, each match in the chain being a TE fragment. Note that a full-length TE copy may correspond to two TE fragments, which, when connected together, correspond to the full TE consensus sequence.

In the previous version of MATCHER, we began by combining the matches found by all three algorithms mentioned above. When two consensus sequences overlapped at a given locus, we retained the sequence with the highest score and truncated the other. We then connected the remaining matches by dynamic programming. In the current version of MATCHER, we first connect the matches by dynamic programming and then filter out overlapping chains of matches. A match that might have been filtered out in the previous version may not be filtered out in the current version, if it is chained with another match, thereby improving fragment connections. TE annotations would be improved by taking into account chains of matches (whole TE copies) rather than TE fragments (single matches).

Once matches are connected by MATCHER, the TEannot pipeline also detects microsatellites by launching and combining the results of TRF [59], Mreps [61] and RepeatMasker. All TE copies are then combined with microsatellite coordinates to filter out short TE matches fully recovered by microsatellites.

In the same spirit, we improved the “long join” procedure. We previously sorted the chains of matches on the basis of length, as a proxy for the age of the TE copies. The rationale behind this was that a TE copy may disappear slowly due to small deletions, becoming shorter over time. We now estimate the age of a TE copy by calculating the ratio of match identity to match length, summing this ratio for all matches in the chain.

Finally, we compared the annotations obtained with *de novo* libraries and reference databanks, by calculating genome coverage and TE copy number, together with sensitivity and specificity, in terms of nucleotide overlaps (Figure S3). A high sensitivity indicates that the annotation based on *de novo* consensus sequences misses few TE nucleotides (false-negatives). A high specificity indicates that the *de novo* annotation identifies few non-TE nucleotides (false-positives).

### Reconstruction of TE families

We compared the patterns of diversification between TE families, by mining the genome with the *de novo* consensus sequences, using BLAT [62] for the rapid identification of well conserved genomic copies. We then constructed a multiple alignment with the TE reference sequence from the public databank, the *de novo* consensus sequence and the genomic copies identified with this sequence (see Figure 4 A–D). For the identification of TE families represented by several *de novo* consensus sequences, we clustered *de novo* consensus sequences with BLASTCLUST from the NCBI-BLAST suite ([63], parameters “-S 0 -L 0.8 -b F -p F”). We then added the best TE reference sequence corresponding to each cluster, and finally built a multiple alignment. The addition of the reference sequence after clustering prevents the *de novo* consensus sequences from being clustered together solely because they overlap with the same reference sequence. This procedure can therefore be used to assist manual curation for newly sequenced genomes without known reference sequences. Multiple alignments were checked by eye, using Jalview [64].

## Supporting Information

Figure S1 **Flow chart of the three first steps of the TEdenovo pipeline.**
(TIF)

Figure S2 **Flow chart of the TEannot pipeline.**
(TIF)

Figure S3 **Comparison of two TE annotations in terms of match boundaries.**
(TIF)

Table S1 **Comparative analysis of self-alignment programs.**
(PDF)

Table S2 **Comparative analysis of clustering programs.**
(PDF)

Table S3 **Comparative analysis of multiple alignment programs.**
(PDF)

Table S4 **Results of the RepeatScout program.**
(PDF)

Table S5 **Results obtained with a combination of several clustering programs in the de novo approach.**
(PDF)

Table S6 **Length parameters used in the TEclassifier program.**
(PDF)

Table S7 **Results of the classification of TE sequences from A. thaliana.**
(PDF)

Table S8 **Consensus sequences matching known genes of D. melanogaster.**
(PDF)

Table S9 **Parameters of the tests for redundancy elimination in TEclassifier.**
(PDF)

Table S10 **Results of coordinate comparisons for TE annotation.**
(PDF)

Table S11 **Comparison of the performances of the RepeatModeler and TEdenovo databanks.**
(PDF)

Table S12 **Comparison of the performances of TE annotation with the databanks of de novo consensus sequences from RepeatModeler and TEdenovo.**
(PDF)

Table S13 **Computation times of the TEdenovo/TEannot pipelines and RepeatScout on the D. melanogaster and A. thaliana genomes.**
(PDF)

Table S14 **Reference sequences entirely retrieved by one clustering method but not by the others, in the D. melanogaster genome.**
(PDF)

Table S15 **Reference sequences entirely retrieved by one clustering method but not by the others, in the A. thaliana genome.**
(PDF)

Table S16 **List of TE families represented by several de novo consensus sequences.**
(PDF)

Table S17 **TE reference sequences for which no “knowledge-based” consensus could be built.**
(PDF)

Table S18 **Comparison of “knowledge-based” libraries with reference databanks.**
(PDF)
