# Supplementary figures and images for: Considering Transposable Element Diversification in De Novo Annotation Approaches
Source: PLoS One. 2011 Jan 31;6(1):e16526. doi: 10.1371/journal.pone.0016526 (PMC3031573; doi:10.1371/journal.pone.0016526)

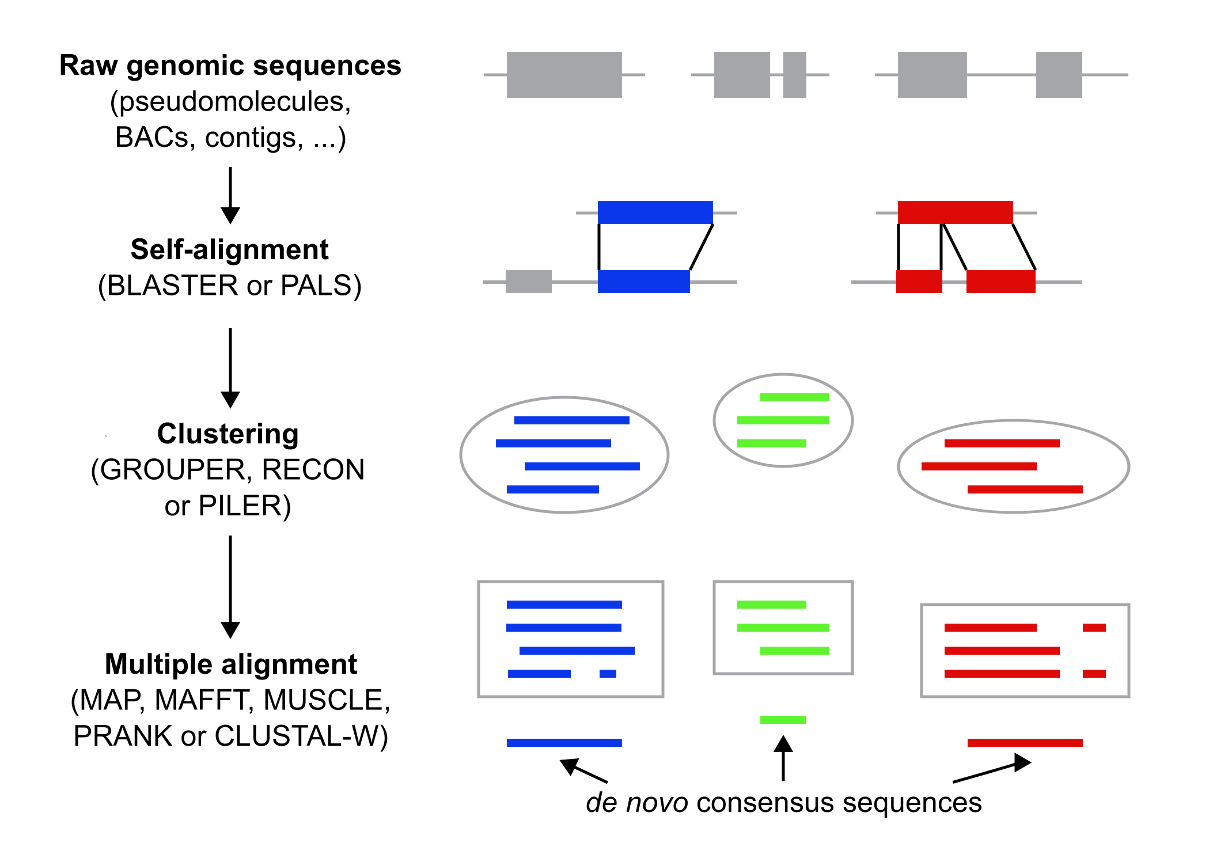

Supplement: Figure S1 — Flow chart of the three first steps of the TEdenovo pipeline. (TIF) [file pone.0016526.s001.tif]

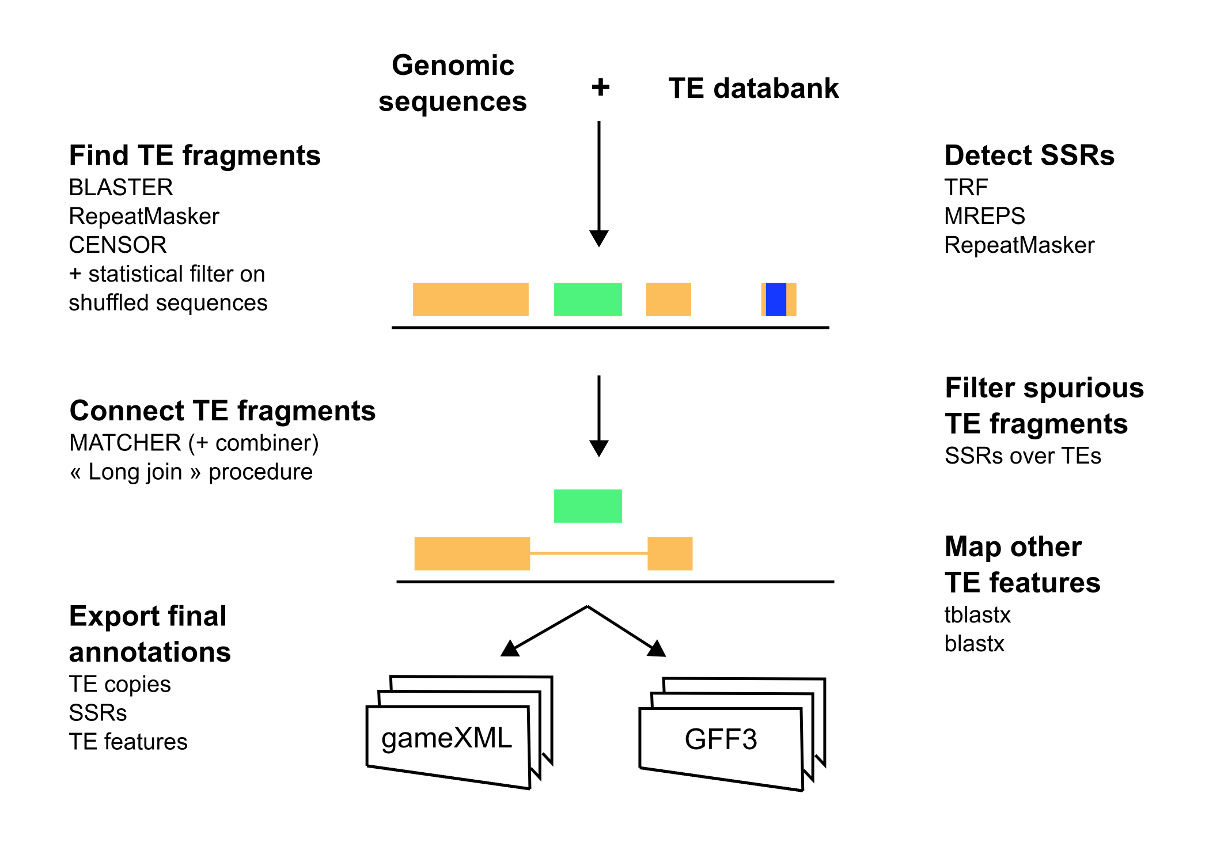

Supplement: Figure S2 — Flow chart of the TEannot pipeline. (TIF) [file pone.0016526.s002.tif]

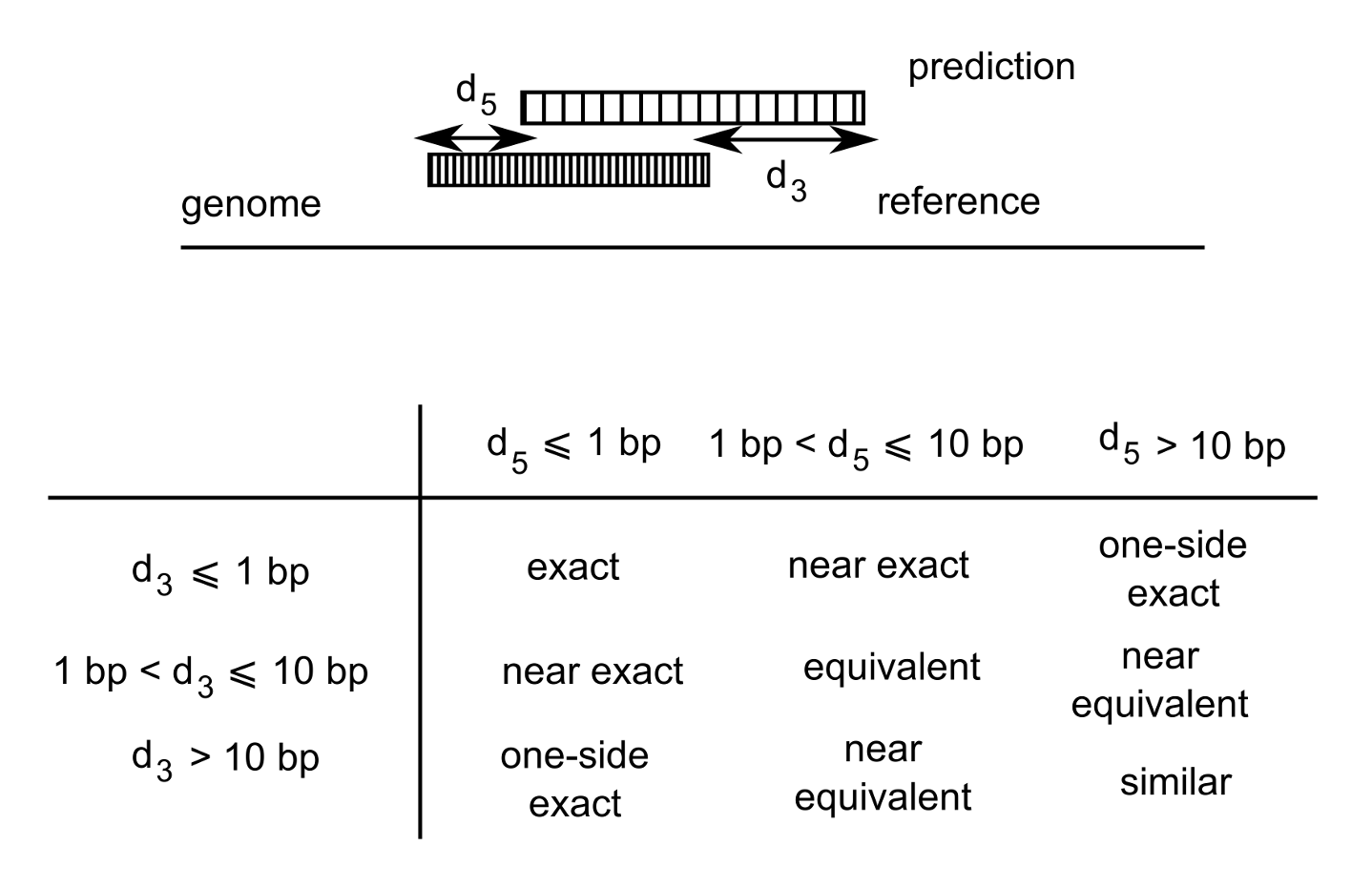

Supplement: Figure S3 — Comparison of two TE annotations in terms of match boundaries. (TIF) [file pone.0016526.s003.tif]
