# Supplementary material for: Considering Transposable Element Diversification in De Novo Annotation Approaches
Source: PLoS One. 2011 Jan 31;6(1):e16526. doi: 10.1371/journal.pone.0016526 (PMC3031573; doi:10.1371/journal.pone.0016526)
Supplement: Table S1 — Comparative analysis of self-alignment programs. (PDF) [file pone.0016526.s004.pdf]

Table S1: Comparative analysis of self-alignment programs

Genome	Genome length (bp)	Program	Number of matches	Length on the genome (bp)	Genome coverage
D. mel.	129,919,500	BLASTER	109,882	9,636,659	7.41%
		PALS	105,059	9,590,737	7.38%
A. tha.	119,146,348	BLASTER	103,728	16,063,506	13.48%
		PALS	51,023	12,547,315	10.53%
