# Supplementary material for: Considering Transposable Element Diversification in De Novo Annotation Approaches
Source: PLoS One. 2011 Jan 31;6(1):e16526. doi: 10.1371/journal.pone.0016526 (PMC3031573; doi:10.1371/journal.pone.0016526)
Supplement: Table S2 — Comparative analysis of clustering programs. (PDF) [file pone.0016526.s005.pdf]

Table S2: Comparative analysis of clustering programs

Genome	Self-alignment	Clustering	Number of clusters	CV of sequence length (mean +- sd)
D. mel.	BLASTER	GROUPER	730	0.463 +- 0.190
		RECON	451	0.711 +- 0.181
		PILER	120	0.566 +- 0.172
	PALS	GROUPER	542	0.466 +- 0.193
		RECON	485	0.742 +- 0.203
		PILER	106	0.567 +- 0.166
A. tha.	BLASTER	GROUPER	1428	0.509 +- 0.184
		RECON	1021	0.702 +- 0.186
		PILER	300	0.603 +- 0.146
	PALS	GROUPER	912	0.519 +- 0.182
		RECON	1000	0.713 +- 0.175
		PILER	242	0.631 +- 0.130

Each cluster contains various genomic sequences, which may differ in length. We therefore calculated the coefficient of variation (CV) of sequence length for each cluster. This coefficient, corresponding to the standard deviation divided by the mean, assesses the dispersion of a distribution. It is high in clusters with sequences of very different lengths, and low in clusters with sequences of similar lengths. We first calculated this coefficient for all the clusters obtained with each method. We then calculated the mean and standard deviation of these coefficients of variation between clusters. As shown in the right column of table S2, on average, RECON clusters are more heterogeneous in terms of sequence length (mean CV > 0.702) than clusters from GROUPER (mean CV < 0.519) and PILER (mean CV < 0.631).
