# Supplementary material for: Considering Transposable Element Diversification in De Novo Annotation Approaches
Source: PLoS One. 2011 Jan 31;6(1):e16526. doi: 10.1371/journal.pone.0016526 (PMC3031573; doi:10.1371/journal.pone.0016526)
Supplement: Table S3 — Comparative analysis of multiple alignment programs. (PDF) [file pone.0016526.s006.pdf]

Table S3: Comparative analysis of multiple alignment programs

Genome	Self-alignment	Clustering	Multiple alignment	S_n*	S_p*	R_{CC}
D. mel.	BLASTER	GROUPER	MAP	80.34%	85.89%	66.20%
		RECON		92.31%	73.17%	66.20%
		PILER		62.39%	84.17%	51.50%
		GROUPER	CLUSTAL-W	80.34%	85.89%	66.20%
		RECON		91.45%	72.06%	20.60%
		PILER		62.39%	84.17%	51.50%
		GROUPER	MAFFT	78.63%	85.89%	64.70%
		RECON		92.31%	73.17%	54.41%
		PILER		62.39%	84.17%	51.50%
		GROUPER	PRANK	80.34%	85.89%	66.20%
		RECON		92.31%	72.95%	61.80%
		PILER		62.39%	84.17%	51.50%
A. tha.	BLASTER	GROUPER	MAP	60.33%	82.42%	39.00%
		RECON		73.77%	61.70%	43.50%
		PILER		47.21%	57.33%	32.45%
		GROUPER	CLUSTAL-W	60.00%	82.42%	38.30%
		RECON		73.11%	60.33%	29.20%
		PILER		47.21%	57.33%	32.45%
		GROUPER	MAFFT	60.00%	82.42%	39.00%
		RECON		74.01%	61.21%	40.25%
		PILER		47.54%	57.33%	32.45%
		GROUPER	PRANK	60.00%	82.42%	39.00%
		RECON		73.77%	61.61%	39.00%
		PILER		47.21%	57.33%	31.80%

S_n*: percentage of “knowledge-based” consensus sequences matching a *de novo* consensus sequence

S_p*: percentage of *de novo* consensus sequences matching a “knowledge-based” consensus sequence

R_{CC}: percentage of fully recovered “knowledge-based” consensus sequences
