# Supplementary material for: Considering Transposable Element Diversification in De Novo Annotation Approaches
Source: PLoS One. 2011 Jan 31;6(1):e16526. doi: 10.1371/journal.pone.0016526 (PMC3031573; doi:10.1371/journal.pone.0016526)
Supplement: Table S4 — Results of the RepeatScout program. (PDF) [file pone.0016526.s007.pdf]

Table S4: Results of the RepeatScout program

Genome	k-mers	Consensus	Mean length (median)	S_n*	S_p*	R_{CC}
D. mel.	5,624,530	1,770	552 (209)	94.87%	57.63%	25.00%
A. tha.	7,569,031	3,417	498 (221)	82.95%	39.54%	13.00%

S_n*: percentage of "knowledge-based" consensus sequences matching a *de novo* consensus sequence

S_p*: percentage of *de novo* consensus sequences matching a "knowledge-based" consensus sequence

R_{CC}: percentage of fully recovered "knowledge-based" consensus sequences
