# Supplementary material for: Considering Transposable Element Diversification in De Novo Annotation Approaches
Source: PLoS One. 2011 Jan 31;6(1):e16526. doi: 10.1371/journal.pone.0016526 (PMC3031573; doi:10.1371/journal.pone.0016526)
Supplement: Table S5 — Results obtained with a combination of several clustering programs in the de novo approach. (PDF) [file pone.0016526.s008.pdf]

## Table S5: Results obtained with a combination of several clustering programs in the *de novo* approach

| Genome | Combination of clustering programs | Consensus sequences | $S_n$* | $S_p$* | $R_{CC}$ |
|---|---|---|---|---|---|
| *D. mel.* | GROUPER | 730 | 80.34% | 85.89% | 66.20% |
| | RECON | 451 | 92.31% | 73.17% | 66.20% |
| | PILER | 120 | 62.39% | 84.17% | 51.50% |
| | GROUPER + RECON | 1181 | 93.16% | 81.03% | 79.40% |
| | GROUPER + PILER | 850 | 81.20% | 85.65% | 66.20% |
| | RECON + PILER | 571 | 92.31% | 75.48% | 73.50% |
| | GROUPER + RECON + PILER | 1301 | 93.16% | 81.32% | 79.40% |
| *A. tha.* | GROUPER | 1428 | 60.33% | 82.42% | 39.00% |
| | RECON | 1021 | 73.77% | 61.70% | 43.50% |
| | PILER | 300 | 47.21% | 57.33% | 32.45% |
| | GROUPER + RECON | 2449 | 74.43% | 73.79% | 49.35% |
| | GROUPER + PILER | 1728 | 63.93% | 78.07% | 39.60% |
| | RECON + PILER | 1321 | 74.10% | 60.71% | 44.15% |
| | GROUPER + RECON + PILER | 2749 | 74.43% | 72.00% | 49.35% |

$S_n$*: percentage of “knowledge-based” consensus sequences matching a *de novo* consensus

$S_p$*: percentage of *de novo* consensus sequences matching a “knowledge-based” consensus

$R_{CC}$: percentage of fully recovered “knowledge-based” consensus sequences
