# Supplementary material for: Considering Transposable Element Diversification in De Novo Annotation Approaches
Source: PLoS One. 2011 Jan 31;6(1):e16526. doi: 10.1371/journal.pone.0016526 (PMC3031573; doi:10.1371/journal.pone.0016526)
Supplement: Table S6 — Length parameters used in the TEclassifier program. (PDF) [file pone.0016526.s009.pdf]

Table S6: Length parameters used in the TEclassifier program

Category	Threshold	Parameter value (bp)
Complete LTR retrotransposon	Maximum length	18,000
	Minimum length	3,900
Complete LINE retrotransposon	Maximum length	13,500
	Minimum length	3,400
Complete TIR DNA-transposon	Maximum length (*)	5,000
	Minimum length	900
SINE	Maximum length	500
MITE	Maximum length	500

(*) For *A. thaliana,* we used 13,000 bp for the maximum length of a complete TIR DNA-transposon.
