# Supplementary material for: Considering Transposable Element Diversification in De Novo Annotation Approaches
Source: PLoS One. 2011 Jan 31;6(1):e16526. doi: 10.1371/journal.pone.0016526 (PMC3031573; doi:10.1371/journal.pone.0016526)
Supplement: Table S7 — Results of the classification of TE sequences from A. thaliana. (PDF) [file pone.0016526.s010.pdf]

Table S7: Results of the classification of TE sequences from *A. thaliana*

Classification	Reference TEs from Repbase	*De novo* consensus sequences (with redundancy)	*De novo* consensus sequences (without redundancy)
Class I "complete" LTR retrotransposon	121	51	25
Class I "incomplete" LTR retrotransposon	16	400	181
Class I "complete" LINE	6	9	6
Class I "incomplete" LINE	3	31	18
Class I SINE	4	0	0
Class II "complete" TIR transposon	16	14	7
Class II "incomplete" TIR transposon	46	164	99
Class II MITE	11	12	6
Helitron	5	15	11
SSR	0	8	8
Confused	0	6	4
No category	88	2039	910
Host genes	2	0	0
Total	318	2749	1275
