# Supplementary material for: Considering Transposable Element Diversification in De Novo Annotation Approaches
Source: PLoS One. 2011 Jan 31;6(1):e16526. doi: 10.1371/journal.pone.0016526 (PMC3031573; doi:10.1371/journal.pone.0016526)
Supplement: Table S8 — Consensus sequences matching known genes of D. melanogaster. (PDF) [file pone.0016526.s011.pdf]

Table S8: Consensus sequences matching known genes of *D. melanogaster*

Of the non-redundant, *de novo* consensus sequences from the *D. melanogaster* genome, 11 overlapped host transcripts over at least 95% of their length, with identity levels exceeding 90%.

#	Clustering method (cluster identifier)	All-by-all matches	Length (bp)	Transcript name	Gene name	Additional information
1	GROUPER (42)	3	454	FBtr0072628	Lysozyme B	
2	GROUPER (45)	20	613	FBtr0089196	Kif3C	See Deloger *et al.* Gene 2009
3	GROUPER (521)	4	2532	FBtr0082512	Hsp70Aa	6 Hsp70 genes are present in the genome.
4	GROUPER (607)	3	3524	FBtr0074205	Mucin 14A	Predicted CDS is composed of a repeat domain (125+ repeat units) of 95 amino acids.
5	GROUPER (709)	4	2778	FBtr0072849	sallimus	3 matches not merged by Grouper.
6	GROUPER (771)	3	3665	FBtr0076140	Mucin 68Ca	Protein sequence contains multiple repeats homologous to Salivary glue proteins. 3 matches not merged by Grouper.
7	GROUPER (825)	6	1442	FBtr0077393	Salivary gland secretion 1	Internal highly repetitive region from gene prediction.
8	PILER (110.3)	3	1133	FBtr0082785	Actin 87E	6 actin genes are present in the genome.
9	RECON (26)	3	2022	FBtr0091706	Muscle-specific protein 300	3 matches on the same chunk
10	RECON (302)	6	5238	FBtr0076820	CG32377	87-aa repeat (6 matches on the same chunk)
11	RECON (45)	20	1167	FBtr0089196	Kif3C	See #2 above.
