# Supplementary material for: Considering Transposable Element Diversification in De Novo Annotation Approaches
Source: PLoS One. 2011 Jan 31;6(1):e16526. doi: 10.1371/journal.pone.0016526 (PMC3031573; doi:10.1371/journal.pone.0016526)
Supplement: Table S9 — Parameters of the tests for redundancy elimination in TEclassifier. (PDF) [file pone.0016526.s012.pdf]

Table S9: Parameters of the tests for redundancy elimination in TEclassifier

Genome	Redundancy elimination	Consensus	S_n*	S_p*	R_{CC}
D. mel.	With redundancy	1301	93.16%	81.32%	79.41%
	95%-98%	593	92.31%	75.72%	77.94%
	90%-90%	494	92.31%	71.86%	70.58%
	80%-80%	428	91.45%	70.33%	70.58%
A. tha.	With redundancy	2749	74.43%	71.99%	49.35%
	95%-98%	1275	74.43%	66.75%	49.35%
	90%-90%	1005	73.44%	61.59%	45.45%
	80%-80%	836	73.44%	57.66%	42.85%

For *D. melanogaster*, the elimination of redundancy with the parameters "90-90" or "80-80" resulted in the loss of six *de novo* consensus sequences that fully recovered "knowledge-based" consensus sequences. These sequences belonged to the 412, invader3, roo, springer, Stalker and Stalker4 families.

For *A. thaliana*, the elimination of redundancy with the parameters "90-90" and "80-80" resulted in the loss of, respectively, six and ten *de novo* consensus sequences that fully recovered "knowledge-based" consensus sequences. These sequences belonged to the ATCOPIA49, ATHILA4B, ATHILA4D, ATREP10, ATREP10A and VANDAL2 (for parameters "90-90"), as well as ATDNA2T9A, ATREP14, ATREP2 and ATREP7 families (for parameters "80-80").

The remaining *de novo* consensus that matched these families were longer than the "knowledge-based" consensus. They contained insertions not present in the "knowledge-based" consensus.
