# Supplementary material for: Considering Transposable Element Diversification in De Novo Annotation Approaches
Source: PLoS One. 2011 Jan 31;6(1):e16526. doi: 10.1371/journal.pone.0016526 (PMC3031573; doi:10.1371/journal.pone.0016526)
Supplement: Table S10 — Results of coordinate comparisons for TE annotation. (PDF) [file pone.0016526.s013.pdf]

Table S10: Results of coordinate comparisons for TE annotation

We compared the annotations between the various combinations of *de novo* libraries and reference databanks, in terms of match coordinates. Several cases can be distinguished on the basis of the distance between the 5' (or 3') coordinate of the test match and that of the reference match (figure S3): distance less than or equal to 1 nt, distance strictly greater than1 nt but less than or equal to 10 nt, and distance strictly greater than 10 nt.

Genome	Prediction	GROUPER	RECON	PILER	G+R+P
D. mel.	Exact	10975	6438	8084	8124
	Near exact	3553	2817	2794	3388
	Equivalent	890	825	789	972
	Near equivalent	5683	5839	5321	6774
	One-side exact	10169	7493	7429	8903
	Similar	18606	19026	15440	21502
	New TE	11077	8864	6874	13533
A. tha.	Exact	3423	4448	2346	3986
	Near exact	2590	2672	2171	2706
	Equivalent	917	916	927	1051
	Near equivalent	8207	8138	7020	7992
	One-side exact	8671	9626	6242	8716
	Similar	36651	38690	30977	36139
	New TE	8614	15482	6588	13023

Same data, but in percentages.

Number of TE fragments:

D. melanogaster: G=43699; R=33072 ; P=32789; GRP=42857

A. thaliana: G=41791; R=49470; P=34818; GRP=44059

**Genome**	**Case**	**Prediction**	**GROUPER**	**RECON**	**PILER**	**G+R+P**
D. mel.	1-to-1	Total 1-to-1	57.34%	58.54%	64.07%	54.98%
		Exact	27.84%	26.07%	27.57%	25.60%
		Near exact	9.68%	9.23%	8.84%	9.32%
		One-side exact	21.72%	20.18%	19.56%	20.35%
		Equivalent	2.27%	2.37%	2.36%	2.53%
		Near equivalent	12.16%	13.49%	13.31%	13.47%
		Similar	26.34%	28.67%	28.36%	28.73%
	1-to-0	New TE	24.80%	22.45%	18.58%	27.65%
	1-to-n	Chimera	17.86%	19.01%	17.34%	17.37%
A. tha.	1-to-1	Total 1-to-1	53.96%	48.10%	54.87%	47.69%
		Exact	11.78%	14.27%	9.19%	14.67%
		Near exact	6.38%	6.40%	5.98%	7.16%
		One-side exact	19.16%	19.35%	16.53%	19.29%
		Equivalent	1.67%	1.75%	1.90%	2.18%
		Near equivalent	14.49%	13.54%	14.37%	14.53%
		Similar	46.52%	44.68%	52.03%	42.17%
	1-to-0	New TE	18.38%	27.46%	16.86%	25.95%
	1-to-n	Chimera	27.66%	24.44%	28.28%	26.35%
