# Supplementary material for: Considering Transposable Element Diversification in De Novo Annotation Approaches
Source: PLoS One. 2011 Jan 31;6(1):e16526. doi: 10.1371/journal.pone.0016526 (PMC3031573; doi:10.1371/journal.pone.0016526)
Supplement: Table S11 — Comparison of the performances of the RepeatModeler and TEdenovo databanks. (PDF) [file pone.0016526.s014.pdf]

Table S11: Comparison of the performances of the RepeatModeler and TEdenovo databanks

Genome	Pipeline	Consensus	S_n*	S_p*	R_{CC}
D. mel.	RepeatModeler	141	77.78%	75.89%	19.11%
	TEdenovo	593	92.31%	75.72%	77.94%
A. tha.	RepeatModeler	177	45.90%	57.63%	5.19%
	TEdenovo	1351	74.43%	66.75%	49.35%

S_n*: percentage of "knowledge-based" consensus sequences matching a *de novo* consensus

S_p*: percentage of *de novo* consensus sequences matching a "knowledge-based" consensus

R_{CC}: percentage of fully recovered "knowledge-based" consensus sequences
