# Supplementary material for: Considering Transposable Element Diversification in De Novo Annotation Approaches
Source: PLoS One. 2011 Jan 31;6(1):e16526. doi: 10.1371/journal.pone.0016526 (PMC3031573; doi:10.1371/journal.pone.0016526)
Supplement: Table S12 — Comparison of the performances of TE annotation with the databanks of de novo consensus sequences from RepeatModeler and TEdenovo. (PDF) [file pone.0016526.s015.pdf]

Table S12: Comparison of the performances of TE annotation with the databanks of *de novo* consensus sequences from RepeatModeler and TEdenovo

Genome	TE library	Consensus	TE genome coverage	Number of copies	S_n	S_p
D. mel.	BDGP	125	10.51%	31208	NA	NA
	TEdenovo	568	11.98%	42847	91.43%	97.35%
	RepeatModeler	141	9.80%	28789	80.18%	98.47%
A. tha.	Repbase	318	19.02%	41146	NA	NA
	TEdenovo	1232	22.77%	44059	87.03%	92.32%
	RepeatModeler	175	15.14%	35432	65.14%	96.60%

S_n : sensitivity = true positives / (true positives + false negatives)

S_p : specificity = true negatives / (true negatives + false positives)
