# Supplementary material for: Considering Transposable Element Diversification in De Novo Annotation Approaches
Source: PLoS One. 2011 Jan 31;6(1):e16526. doi: 10.1371/journal.pone.0016526 (PMC3031573; doi:10.1371/journal.pone.0016526)
Supplement: Table S13 — Computation times of the TEdenovo/TEannot pipelines and RepeatScout on the D. melanogaster and A. thaliana genomes. (PDF) [file pone.0016526.s016.pdf]

Table S13: Computation times of the TEdenovo/TEannot pipelines and RepeatScout on the *D. melanogaster* and *A. thaliana* genomes

Genome	Step	Running time
D. melanogaster	TEdenovo 1 BLASTER (138 jobs)	3m (per job)
	TEdenovo 2 GROUPER	51s
	TEdenovo 2 RECON	15m
	TEdenovo 2 PILER	21s
	TEdenovo 3 MAP (after GROUPER, 730 jobs)	48s (per job)
	TEdenovo 3 MAP (after RECON, 451 jobs)	17s (per job)
	TEdenovo 3 MAP (after PILER, 120 jobs)	45s (per job)
	TEdenovo 4 (TEclassifier step 1, 651 jobs)	2m (per job)
	TEdenovo 5 (TEclassifier step 2)	1h 16m
D. melanogaster	REPEATSCOUT (2 steps)	43m
D. melanogaster	TEannot 1 Preprocessing of input data	2m
	TEannot 2 BLASTER (138 jobs)	2m 20s (per job)
	TEannot 2 REPEATMASKER (138 jobs)	2m 6s (per job)
	TEannot 2 CENSOR (138 jobs)	1m 10s (per job)
	TEannot 2 BLASTER (random, 138 jobs)	1m 29s (per job)
	TEannot 2 REPEATMASKER (random, 138 jobs)	1m 32s (per job)
	TEannot 2 CENSOR (random, 138 jobs)	56s (per job)
	TEannot 3 Combine TE annotations	1m 40s
	TEannot 4 TandemRepeatFinder (138 jobs)	5s (per job)
	TEannot 4 MREPS (138 jobs)	4s (per job)
	TEannot 4 REPEATMASKER-SSR (138 jobs)	33s (per job)
	TEannot 5 Combine SSR annotations	10m
	TEannot 7 Postprocessing of TE annotations	6m 30s
	TEannot 8 export annotations (gameXML)	3m
A. thaliana	TEdenovo 1 BLASTER (126 jobs)	3m 3s (per job)
	TEdenovo 2 GROUPER	51s
	TEdenovo 2 RECON	26m 37s
	TEdenovo 2 PILER	13s
	TEdenovo 3 MAP (after GROUPER, 1428 jobs)	26s (per job)
	TEdenovo 3 MAP (after RECON, 1021 jobs)	10s (per job)
	TEdenovo 3 MAP (after PILER, 300 jobs)	3s (per job)
	TEdenovo 4 (TEclassifier step 1, 1419 jobs)	2m 22s (per job)
	TEdenovo 5 (TEclassifier step 2)	1h 30m
A. thaliana	REPEATSCOUT	43min

Genome	Step	Running time
A. thaliana	TEannot 1 Preprocessing of input data	1m 10s
	TEannot 2 BLASTER (126 jobs)	10m 35s (per job)
	TEannot 2 REPEATMASKER (126 jobs)	4m 24s (per job)
	TEannot 2 CENSOR (126 jobs)	4m 24s (per job)
	TEannot 2 BLASTER (random, 126 jobs)	6m 19s (per job)
	TEannot 2 REPEATMASKER (random, 126 jobs)	3m 21s (per job)
	TEannot 2 CENSOR (random, 126 jobs)	3m 24s (per job)
	TEannot 3 Combine TE annotations	5m 28s
	TEannot 4 TandemRepeatFinder (126 jobs)	5s (per job)
	TEannot 4 MREPS (126 jobs)	4s (per job)
	TEannot 4 REPEATMASKER-SSR (126 jobs)	29s (per job)
	TEannot 5 Combine SSR annotations	7m 29s
	TEannot 7 Postprocessing of TE annotations	6m 34s
	TEannot 8 export annotations (gameXML)	3m 33s

For each step in which jobs are launched in parallel, we indicate the mean running time per job.

For the step 5 of the TEdenovo pipeline, we indicated an average estimate of the total running time. In fact, in this step, when removing redundancy among consensus, all computations are done in parallel, but it would have been too long to detail all the parallelized sub-steps.

For RepeatScout, we give the sum of the running times of the “build_lmer_table” and “RepeatScout” binaries.

We obtained these computation times using the computer cluster from the GenOuest platform (http://genouest.org/). We used the queues “sgi64g.q” and “sgi144g.q” which have respectively 11 machines, each with 8 cores and 64G RAM, and 7 machines, each with 16 cores and 144G RAM. The step 2 of TEdenovo (clustering of all-by-all matches) is the step that requires the most RAM. However, although the RAM of the computer cluster from the GenOuest plateform is pretty big for each machine, the step 2 doesn't need as much as 64G or 144G RAM. Indeed, we ran it successfully on another computer cluster having only 4G of RAM per node.

Moreover, it is important to note that, in each step of the TEdenovo and TEannot pipelines, one or several programs and scripts are submitted to a cluster node using the SGE batch-queuing system. Therefore, even if a job only needs a few minutes in average to be completed, the running time of the whole step can be much longer. Indeed, the running time strongly depends on the load of the computer cluster (i.e. the number of jobs submitted by other users, and their respective duration).
