# Supplementary material for: Considering Transposable Element Diversification in De Novo Annotation Approaches
Source: PLoS One. 2011 Jan 31;6(1):e16526. doi: 10.1371/journal.pone.0016526 (PMC3031573; doi:10.1371/journal.pone.0016526)
Supplement: Table S14 — Reference sequences entirely retrieved by one clustering method but not by the others, in the D. melanogaster genome. (PDF) [file pone.0016526.s017.pdf]

Table S14: Reference sequences entirely retrieved by one clustering method but not by the others, in the *D. melanogaster* genome

Family	Classification	Length of the reference sequence	Number of full-length fragments	Number of full-length copies	Clustering method retrieving the entire reference sequence	Results of the other clustering methods
invader3	Class I LTR	5484	3	4	GROUPER	R:CI / P:NA
invader4	Class I LTR	3105	1	2	GROUPER	R:II / P:IC
Stalker	Class I LTR	7256	3	3	GROUPER	R:II / P:IC
Stalker4	Class I LTR	7359	2	3	GROUPER	R:II / P:IC
1360	Class II TIR	3409	2	2	RECON	G:IC / P:IC
Doc2-element	Class I LINE	4789	1	3	RECON	G:IC / P:II
G2	Class I LINE	3102	4	4	RECON	G:IC / P:II
gypsy4	Class I LTR	6852	0	3	RECON	G:IC / P:NA
Max-element	Class I LTR	8556	2	2	RECON	G:IC / P:NA
R1A1-element	Class I LINE	5356	2	2	RECON	G:IC / P:NA
rover	Class I LTR	7318	2	2	RECON	G:IC / P:II
Tabor	Class I LTR	7345	2	2	RECON	G:IC / P:II
Tc1-2	Class II TIR	1644	1	1	RECON	G:IC / P:NA

In the column on the far right, “G” stands for GROUPER, “R” for RECON and “P” for PILER. “CI” indicates that the reference sequence matches completely (over more than 95% of its length) a *de novo* consensus, which matches incompletely (over less than 95% of its length). “IC” means that the reference sequence matches incompletely whereas the *de novo* consensus matches completely. “II” indicates that both sequences match incompletely. “NA” indicates that the reference sequence matches none of the *de novo* consensus sequences.
