# Supplementary material for: Considering Transposable Element Diversification in De Novo Annotation Approaches
Source: PLoS One. 2011 Jan 31;6(1):e16526. doi: 10.1371/journal.pone.0016526 (PMC3031573; doi:10.1371/journal.pone.0016526)
Supplement: Table S15 — Reference sequences entirely retrieved by one clustering method but not by the others, in the A. thaliana genome. (PDF) [file pone.0016526.s018.pdf]

Table S15: Reference sequences entirely retrieved by one clustering method but not by the others, in the *A. thaliana* genome

Family	Classification	Length of the reference sequence (bp)	Number of full-length fragments	Number of full-length copies	Clustering method retrieving the entire reference sequence	Results of the other clustering methods
ATDNA1T9A	Class II TIR	3552	3	3	GROUPER	R:IC / P:II
ATENSPM5	Class II TIR	8717	0	4	GROUPER	R:IC / P:NA
ATGP2N	Class I LTR	5233	0	2	GROUPER	R:IC / P:II
ATGP6	Class I LTR	5695	0	1	GROUPER	R:IC / P:II
ATHILA	Class I LTR	10492	2	3	GROUPER	R:II / P:II
ATHILA2	Class I LTR	10923	4	5	GROUPER	R:II / P:IC
ATHILA6A	Class I LTR	11611	1	1	GROUPER	R:II / P:IC
VANDAL15	Class II TIR	5332	1	1	GROUPER	R:IC / P:NA
ARNOLD2	Class II TIR	15490	2	2	RECON	G:IC / P:NA
ATCOPIA1	Class I LTR	5139	2	2	RECON	G:NA / P:II
ATCOPIA10	Class I LTR	5186	3	3	RECON	G:NA / P:NA
ATCOPIA31	Class I LTR	4626	2	2	RECON	G:NA / P:NA
ATCOPIA49	Class I LTR	5220	5	6	RECON	G:II / P:NA
ATDNAI27T9B	Class II TIR	2090	2	2	RECON	G:IC / P:II
ATENSPM6	Class II TIR	8825	2	9	RECON	G:IC / P:NA
ATGP2	Class I LTR	7623	4	6	RECON	G:IC / P:II
ATGP5	Class I LTR	6359	1	2	RECON	G:IC / P:NA
ATREP14	Class II Helitron	737	11	12	RECON	G:IC / P:II
ATREP16	Class II TIR	1391	2	2	RECON	G:NA / P:NA
BRODYAGA1	Class II TIR	1184	8	8	RECON	G:II / P:II
LIMPET1	Class II TIR	1874	2	2	RECON	G:IC / P:II
SIMPLEHAT1	Class II TIR	1059	4	4	RECON	G:IC / P:NA

Family	Classification	Length of the reference sequence (bp)	Number of full-length fragments	Number of full-length copies	Clustering method retrieving the entire reference sequence	Results of the other clustering methods
VANDAL 2	Class II TIR	15253	3	6	RECON	G:IC / P:IC

In the column on the far right, “G” stands for GROUPER, “R” for RECON and “P” for PILER. “CI” indicates that the reference sequence matches completely (over more than 95% of its length) a *de novo* consensus, which matches incompletely (over less than 95% of its length). “IC” means that the reference sequence matches incompletely whereas the *de novo* consensus matches completely. “II” indicates that both sequences match incompletely. “NA” indicates that the reference sequence matches none of the *de novo* consensus sequences.
