# Supplementary material for: Considering Transposable Element Diversification in De Novo Annotation Approaches
Source: PLoS One. 2011 Jan 31;6(1):e16526. doi: 10.1371/journal.pone.0016526 (PMC3031573; doi:10.1371/journal.pone.0016526)
Supplement: Table S16 — List of TE families represented by several de novo consensus sequences. (PDF) [file pone.0016526.s019.pdf]

Table S16: List of TE families represented by several *de novo* consensus sequences

Genome	Reference sequence(s)	Consensus from GROUPER	Consensus from RECON	Consensus from PILER
D. mel.	Stalker, Stalker2 and Stalker4	15	3	0
	FB	11	8	0
	invader1	5	1	1
	GATE	3	2	0
	297	3	1	1
	baggins	1	4	0
	invader4	2	2	1
	Idefix	1	2	1
	springer, gypsy3	0	3	0
	mdg1	2	0	1
	micropia	1	2	0
A. tha.	ATHILA6A, ATHILA6B	8	3	0
	SIMPLEHAT2	7	3	1
	ATREP1, ATREP2	5	2	2
	ATGP2N	4	1	0
	ARNOLDY1, ARNOLDY2	2	5	0
	ARNOLD1 to 4	2	5	0
	VANDAL2	3	3	0
	ATLANTYS2	1	4	0
	ATHILA4D	3	2	1
	VANDAL6	2	2	0
	ATREP2A	1	0	4
	HELITRONY2	1	2	0
	ATMUNX1	3	1	0
	ATREP4	2	2	0

These TE families were formed using BLASTCLUST, with a coverage of 80%. Manual curation may result in these families being represented by fewer consensus sequences than indicated.
