# Supplementary material for: Considering Transposable Element Diversification in De Novo Annotation Approaches
Source: PLoS One. 2011 Jan 31;6(1):e16526. doi: 10.1371/journal.pone.0016526 (PMC3031573; doi:10.1371/journal.pone.0016526)
Supplement: Table S17 — TE reference sequences for which no “knowledge-based” consensus could be built. (PDF) [file pone.0016526.s020.pdf]

Table S17: TE reference sequences for which no "knowledge-based" consensus could be built

Genome	Reference sequence	Length (bp)	Comments
D. mel.	Helitron	564	no copy
	BS4	754	less than 3 copies
	Q-element	759	not possible to build a consensus
	Stalker3T	372	no copy more than 100 bp long
	Penelope	804	"knowledge-based" consensus sequence could not identify a genomic copy
	P-element	2907	known to be absent from the sequenced strain
	TART-A	13424	consensus does not match its reference sequence, known to be present at telomeres
	TART-B	10654	consensus does not match its reference sequence, known to be present at telomeres
	TART-C	11124	consensus does not match its reference sequence, known to be present at telomeres
A. tha.	ATCOPIA6	4718	consensus does not match its reference sequence with the parameters used
	ATCOPIA18	2384	only two copies (1 truncated and 1 full-length)
	ATCOPIA30	4237	consensus does not match its reference sequence with the parameters used
	ATCOPIA31A	4664	only two copies (1 truncated and 1 full-length)
	ATCOPIA47	5063	three copies (2 truncated, including 1 less than 100 bp long, 1 full-length)
	ATCOPIA80	4280	only two copies (1 truncated and 1 full-length)
	ATCOPIA84	4882	only two copies (1 truncated and 1 full-length)
	ATCOPIA85	3878	consensus does not match its reference sequence with the parameters used
	ATCOPIA91	5270	five copies (3 less than 100 bp long and 1 full-length)
	ATREP19		consensus does not match its reference sequence with the parameters used
	DRL1	250	only one copy
	TA1_AT	514	only one copy
	TA12	949	consensus does not match its reference sequence with the parameters used
