# Supplementary material for: Considering Transposable Element Diversification in De Novo Annotation Approaches
Source: PLoS One. 2011 Jan 31;6(1):e16526. doi: 10.1371/journal.pone.0016526 (PMC3031573; doi:10.1371/journal.pone.0016526)
Supplement: Table S18 — Comparison of “knowledge-based” libraries with reference databanks. (PDF) [file pone.0016526.s021.pdf]

Table S18: Comparison of “knowledge-based” libraries with reference databanks

Genome	Sequences in the reference databank	Sequences in the “knowledge-based” library	S_n*	S_p*	R_{CC}
D. mel.	126	117	94.44%	100.00%	100.00%
A. tha.	318	309	98.43%	100.00%	100.00%

S_n*: percentage of “knowledge-based” consensus sequences matching a *de novo* consensus sequence

S_p*: percentage of *de novo* consensus sequences matching a “knowledge-based” consensus sequence

R_{CC}: percentage of fully recovered “knowledge-based” consensus sequences
